# Loss of peroxiredoxin-2 exacerbates eccentric contraction-induced force loss in dystrophin-deficient muscle

**DOI:** 10.1038/s41467-018-07639-3

**Published:** 2018-11-30

**Authors:** John T. Olthoff, Angus Lindsay, Reem Abo-Zahrah, Kristen A. Baltgalvis, Xiaobai Patrinostro, Joseph J. Belanto, Dae-Yeul Yu, Benjamin J. Perrin, Daniel J. Garry, George G. Rodney, Dawn A. Lowe, James M. Ervasti

**Affiliations:** 1 Molecular, Cellular, Developmental Biology, and Genetics Graduate Program, University of Minnesota, Minneapolis, MN 55455 USA; 2 Divisions of Rehabilitation Science and Physical Therapy, Department of Rehabilitation Medicine, University of Minnesota, Minneapolis, MN 55455 USA; 3 Department of Molecular Physiology and Biophysics, Baylor College of Medicine, Houston, TX 77030 USA; 4 Department of Biochemistry, Molecular Biology, and Biophysics, University of Minnesota, Minneapolis, MN 55455 USA; 5 Aging Research Center, Korea Research Institute of Bioscience and Biotechnology (KRIBB), Daejeon, Republic of Korea; 6 Department of Biology, Indiana University-Purdue University Indianapolis, Indianapolis, IN 46022 USA; 7 Lillehei Heart Institute and Department of Medicine, University of Minnesota, Minneapolis, MN 55455 USA

## Abstract

Force loss in skeletal muscle exposed to eccentric contraction is often attributed to injury. We show that EDL muscles from dystrophin-deficient *mdx* mice recover 65% of lost force within 120 min of eccentric contraction and exhibit minimal force loss when the interval between contractions is increased from 3 to 30 min. A proteomic screen of *mdx* muscle identified an 80% reduction in the antioxidant peroxiredoxin-2, likely due to proteolytic degradation following hyperoxidation by NADPH Oxidase 2. Eccentric contraction-induced force loss in *mdx* muscle was exacerbated by peroxiredoxin-2 ablation, and improved by peroxiredoxin-2 overexpression or myoglobin knockout. Finally, overexpression of γ_cyto_- or β_cyto_-actin protects *mdx* muscle from eccentric contraction-induced force loss by blocking NADPH Oxidase 2 through a mechanism dependent on cysteine 272 unique to cytoplasmic actins. Our data suggest that eccentric contraction-induced force loss may function as an adaptive circuit breaker that protects *mdx* muscle from injurious contractions.

## Abstract (web summary)

In the mdx mouse model of Duchenne muscular dystrophy, muscle contractions lead to force loss, which is attributed to myofibre damage. Here, the authors show that force loss is instead mediated by a redox circuit involving NOX2, PROX1, myoglobin and cytoplasmic actins, and suggest that it may be a protective mechanism to prevent excessive contraction-induced myofibre damage.

## Introduction

Duchenne muscular dystrophy (DMD) is an X-linked recessive disease caused by deleterious mutations in the *DMD* gene, rendering non-functional forms or complete absence of the protein dystrophin^1^. Dystrophin is normally enriched at subsarcolemmal structures known as costameres, where it links the cortical actin cytoskeleton to the extracellular matrix through interactions with a membrane-bound glycoprotein complex^2,3^. DMD is one of the most common and severe forms of muscular dystrophy, affecting ~1:4000 boys^4^. Dystrophin deficiency leads to progressive weakness and deterioration of skeletal muscle beginning at 3 to 5 years of age. DMD patients typically become non-ambulatory by 12 years of age, with death ensuing by the second or third decade due to cardiac or respiratory failure^5^. Although several treatments exist, including ventilatory support and use of corticosteroids^6,7^, there is no cure for DMD.

Much of the mechanistic understanding of DMD has been elucidated in the dystrophin-deficient *mdx* mouse model, which encodes a nonsense mutation in exon 23 of the *DMD* gene ablating dystrophin protein expression^8,9^. Although the *mdx* mouse presents a milder phenotype compared to DMD patients^10^, several aspects of the disease are recapitulated in the model, including pervasive muscle weakness^11^, substantial histopathology due to repetitive rounds of muscle degeneration and regeneration^12^, and elevated serum creatine kinase levels^13^. Landmark studies by Sweeney and colleagues^14^ and Moens et al.^15^ demonstrated that *mdx* skeletal muscles are particularly sensitive to precipitous loss of contractile function after performing eccentric contractions (ECCs). With validation by many laboratories around the world as a highly robust and reproducible phenotype of murine dystrophy, the measurement of force loss induced by ECCs in *mdx* mice has emerged as an important quantitative readout for the efficacy of potential DMD therapies^16,17^. However, the molecular mechanism by which ECC force loss occurs in *mdx* muscle is poorly understood.

ECC force loss in *mdx* muscle is frequently referred to as contraction-induced “injury” or “damage”, implying that some form of slowly reversible damage has occurred within the myofiber that would take days to fully recover^18^. Recent studies suggest that morphological defects at the neuromuscular junction^19^, loss of sarcolemmal excitability^20^, and myofibrillar dysfunction^21^ all contribute to ECC force loss in *mdx* muscle. Several signaling mechanisms have also been implicated, including calcium^22,23^, Akt/PKB kinase^24^, neuronal nitric oxide synthase^25^, and redox pathways^26^. The effect of reactive oxygen species (ROS) on ECC force loss is particularly interesting considering recent discoveries of aberrant stretch-activated ROS in *mdx* skeletal muscle^27–30^.

In this study, we demonstrate that isolated *mdx* skeletal muscle recovers 65% of lost force production within 120 min of ECC and loses minimal force if the interval between eccentric contractions is increased from 3 to 30 min. To gain a mechanistic understanding of ECC force loss, we employed isobaric tags for relative and absolute quantification (iTRAQ) proteomics to identify proteins that were differentially expressed in skeletal muscle of *mdx* mice overexpressing nonmuscle γ_cyto_-actin (*mdx*/Actg1-TG), which we have previously shown are significantly protected against ECC force loss^31^. We identified the antioxidant enzyme peroxiredoxin-2 (PrxII) as significantly decreased in *mdx* muscle compared to wild type (WT), but restored to WT levels in *mdx*/Actg1-TG mice. We verify that hyperactive NADPH oxidase 2 (NOX2)-dependent ROS production contributes to ECC force loss and likely causes proteolytic degradation of hyperoxidized PrxII. Ablation of PrxII exacerbated ECC force loss in *mdx* muscle, while overexpression of PrxII led to a dose-dependent protection against ECC force loss. We also demonstrate that myoglobin participates in ECC force loss in *mdx* muscle, likely through the production of hydroxyl radicals via Fenton chemistry. We further establish that oxidation-sensitive cysteine 272 unique to γ_cyto_- and β_cyto_-actin is required to protect *mdx* muscle from ECC force loss while blocking NOX2-mediated ROS production. Finally, we show that overexpression of PrxII leads to increased sarcolemmal damage in *mdx* muscle exposed to eccentric contractions in vivo. Together, these data suggest that ECC may drive a transient, redox-based inhibition of contractility that protects dystrophin-deficient muscle from more catastrophic structural damage caused by subsequent high-force contractions.

## Results

### Rapid recovery of ECC force loss in *mdx* skeletal muscle

**Fig. 1:**
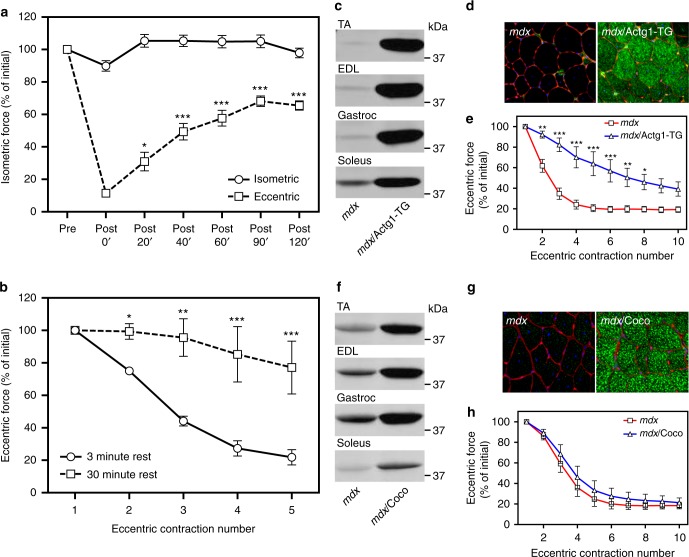
Eccentric contraction-induced force loss in *mdx* muscle recovers rapidly and is partially protected by overexpression of γ_cyto_-actin, but not α_cardiac_-actin. **a** Recovery of isometric force production in isolated extensor digitorum longus (EDL) muscles from *mdx* mice subjected to 10 maximal isometric or eccentric contractions. Values are expressed as a percentage of the isometric force measured before the 10 contractions (Pre) for each timepoint listed; *n* = 4 for both conditions. *P < 0.05, ***P < 0.001 compared with Post 0’; two-way ANOVA. **b** Increasing the time interval between eccentric contractions from 3 to 30 min significantly diminishes the measured force loss in EDL muscles from *mdx* mice; *n* = 4 for both conditions. *P < 0.05, **P < 0.01, ***P < 0.001 compared to 3 min Rest; two-way ANOVA. **c** Immunoblot analysis of γ_cyto_-actin in tibialis anterior (TA), extensor digitorum longus (EDL), gastrocnemius (Gastroc), and soleus muscles from *mdx*/Actg1-TG mice versus non-transgenic *mdx* littermates. **d** Immunofluorescence analysis of γ_cyto_-actin (green), laminin (red), and DAPI (blue) in 10 µm cryosections of quadriceps muscle from *mdx*/Actg1-TG mice versus non-transgenic *mdx* littermates. **e** EDL muscles isolated from *mdx*/Actg1-TG mice and non-transgenic *mdx* littermates were subjected to 10 eccentric contractions and the force measured at each contraction expressed as a percentage of the force produced during the first contraction; *n* = 4 for both genotypes. *P < 0.05, **P < 0.01, ***P < 0.001 compared to *mdx*; two-way ANOVA. **f** Immunoblot analysis of α_ca_-actin in tibialis anterior (TA), extensor digitorum longus (EDL), gastrocnemius (Gastroc), and soleus muscles from *mdx*/Coco mice versus non-transgenic *mdx* littermates. **g** Immunofluorescence analysis of α_ca_-actin (green), laminin (red), and DAPI (blue) in 10 µm cryosections of quadriceps muscle from *mdx*/Coco mice versus non-transgenic *mdx* littermates. **h** EDL muscles isolated from *mdx*/Coco mice and non-transgenic *mdx* littermates were subjected to 10 eccentric contractions and the force measured at each contraction expressed as a percentage of the force produced during the first contraction; *n* = 4 for both genotypes. Throughout, error bars represent means ± SEM

Our standard ECC protocol incorporates a 3 min interval between the 10 ECCs to eliminate fatigue, which is corroborated by the lack of force loss when isolated *mdx* extensor digitorum longus (EDL) muscles perform 10 more energy consumptive isometric contractions^32,33^ (Fig. 1a). Imposing 10 ECCs on the contralateral EDL resulted in the expected 90% drop in force; however, we observed 65% recovery of lost force production within 2 h (Fig. 1a). More surprisingly, the ECC force loss measured in *mdx* EDL muscles was significantly attenuated when the interval between ECC was increased from 3 to 30 min (Fig. 1b). These data demonstrate that dystrophin-deficient *mdx* muscle can rapidly recover from the perturbation imposed by one or multiple ECCs.

### Overexpression of γ_cyto_-actin protects *mdx* muscle from ECC force loss

We previously generated a mouse model that overexpresses γ_cyto_-actin specifically in skeletal muscle (Actg1-TG) to levels that replace 40% of α_skeletal_-actin in myofibrils^34^ (Fig. 1c, d) and which significantly protects *mdx* muscle from ECC force loss^31^ (Fig. 1e). To address whether protection of *mdx* muscle from ECC force loss depends specifically on the γ_cyto_-actin isoform, we crossed transgenic mice (Coco) that overexpress alpha-cardiac actin (α_ca_-actin) specifically in skeletal muscle^35^ onto the *mdx* background (*mdx*/Coco). Similar to the robust expression of γ_cyto_-actin in *mdx*/Actg1-TG (Fig. 1c, d), muscles from *mdx*/Coco animals all showed high α_ca_-actin expression that was uniformly distributed throughout the muscle fibers (Fig. 1f, g). In contrast to *mdx*/Actg1-TG mice (Fig. 1e), however, EDL muscles from *mdx*/Coco animals were not significantly protected from ECC force loss (Fig. 1h). These data led us to conclude that some feature specific to γ_cyto_-actin is necessary to protect *mdx* muscles from ECC force loss.

### Overexpression of γ_cyto_-actin restores PrxII levels in *mdx* muscle

**Fig. 2:**
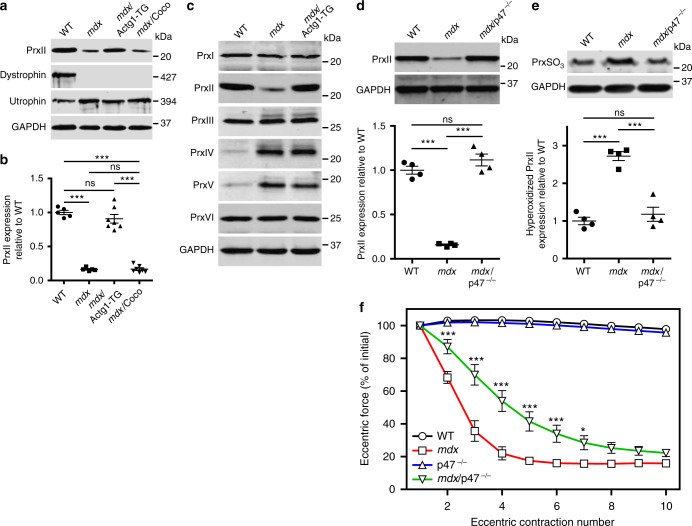
Peroxiredoxin-2 is significantly decreased in *mdx* skeletal muscle and restored by γ_cyto_-actin overexpression and genetic ablation of NOX2 activity. **a** Immunoblot analysis of PrxII, dystrophin, utrophin, and GAPDH in WT, *mdx*, *mdx*/Actg1-TG, and *mdx*/Coco gastrocnemius muscles. **b** Immunoblot quantitation demonstrated that PrxII levels in *mdx* skeletal muscle were 16.5 ± 0.03% of WT and restored in *mdx*/Actg1-TG muscle to levels not different from WT, but not in *mdx*/Coco muscle; *n* = 5 for WT and *mdx;* *n* = 7 for *mdx*Actg1-TG and *mdx*/Coco. ***P < 0.001, ns  no significance; one-way ANOVA. **c** Immunoblot analysis of peroxiredoxins 1–6 in gastrocnemius muscles from WT, *mdx*, and *mdx*/Actg1-TG mice. PrxII was the only peroxiredoxin isoform that was both altered in *mdx* compared to WT, and also restored to its WT level by muscle-specific γ_cyto_-actin overexpression. **d** Immunoblot analysis of PrxII in WT, *mdx*, and *mdx*/p47^–/–^ gastrocnemius muscles demonstrated a restoration of PrxII to WT levels in *mdx*/p47^–/–^ muscle; *n* = 4 for each genotype. ***P < 0.001, ns no significance; one-way ANOVA. **e** Immunoblot analysis demonstrated significantly elevated hyperoxidized peroxiredoxin (PrxSO_3_) in *mdx* compared to WT, and restored to WT levels in *mdx*/p47^–/–^ gastrocnemius muscles; *n* = 4 for each genotype. ***P < 0.001, ns no significance; one-way ANOVA. **f** EDL muscles isolated from WT, *mdx*, p47^–/–^, and *mdx*/p47^–/–^ mice were subjected to 10 eccentric contractions and the forces measured expressed as a percentage of the force generated during the first eccentric contraction; *n* = 4 for WT and *mdx*; *n* = 3 for p47^–/–^; *n* = 7 for *mdx*/p47^–/–^. *P < 0.05, ***P < 0.001 compared to *mdx*; two-way ANOVA. Throughout, error bars represent means ± SEM

To begin to understand how γ_cyto_-actin overexpression protects *mdx* muscle from ECC force loss, we performed 8-plex isobaric tags for relative and absolute quantification (iTRAQ)-based mass spectrometry analysis^36^ on tibialis anterior (TA) muscle lysates isolated from *mdx*/Actg1-TG mice controlled against non-transgenic *mdx* littermates. The iTRAQ screen initially identified 1963 proteins representing all major protein constituents of adult myofibers in both *mdx* and *mdx*/Actg1-TG muscle (Supplementary Data 1). After applying a 1% false discovery rate to the initial protein list, 144 high-confidence proteins survived (Supplementary Data 2), with only 2 proteins demonstrating significantly different levels between *mdx* and *mdx*/Actg1-TG muscles. One differentially expressed protein was γ_cyto_-actin, upregulated 28-fold in *mdx*/Actg1-TG over *mdx*, while the second protein PrxII was significantly upregulated 3.75-fold in *mdx*/Actg1-TG over *mdx*. In addition to confirming the iTRAQ result, western blot analysis with PrxII-specific antibodies demonstrated that PrxII is significantly decreased in *mdx* muscle compared to WT and restored to WT levels in *mdx*/Actg1-TG mice (Fig. 2a, b). In contrast, PrxII levels in *mdx*/Coco muscle overexpressing α_ca_-actin were not different from *mdx* muscle (Fig. 2a, b). PrxII is one member in a family of six sulfhydryl-dependent cellular peroxidases that reduce endogenous hydrogen peroxide (H_2_O_2_)^37^. Western blot analyses for the other five family members revealed that PrxI, PrxIII, and PrxVI levels were not different between WT, *mdx*, and *mdx*/Actg1-TG muscles, while PrxIV and PrxV were substantially elevated in *mdx* and *mdx*/Actg1-TG compared to WT (Fig. 2c). Thus, PrxII was the only peroxiredoxin significantly altered in *mdx* muscle (decreased) and restored to its WT level in *mdx*/Actg1-TG (Fig. 2c).

### NOX2 ablation rescues PrxII levels and attenuates ECC force loss in *mdx* muscle

PrxII mRNA levels were not different between WT, *mdx*, and *mdx*/Actg1-TG muscles (Supplementary Fig. 1a,b), suggesting the loss of PrxII protein in *mdx* muscle is post-transcriptional. Recovery of PrxII levels in *mdx*/Actg1-TG muscle does not seem to involve a direct interaction between PrxII and γ_cyto_-actin, as PrxII did not bind to F- or G-actin in vitro (Supplementary Fig. 1c-f) or in vivo (Supplementary Fig. 1g). Three groups recently demonstrated increased expression of NOX2 subunits in *mdx* muscle, which were shown to produce significantly more ROS (ultimately in the form of H_2_O_2_) in response to mechanical stretch^27,29,30^. Because pharmacological inhibition of NOX2 by apocynin was also shown to protect *mdx* muscle from ECC force loss^27^, we performed experiments to elucidate the relationship between NOX2 and PrxII in *mdx* muscle. We confirmed that several NOX2 subunits (gp91^phox^, p67^phox^, p22^phox^, and Rac1) were increased in *mdx* muscle and show that levels remained elevated in *mdx*/Actg1-TG muscle (Supplementary Fig. 2a). We next compared PrxII protein levels in WT, *mdx*, and *mdx*/p47^–/–^ muscle, which is ablated for the p47^phox^ subunit necessary for NOX2 activity^38^. PrxII was restored to WT levels in *mdx*/p47^–/–^ muscle (Fig. 2d), supporting a role for NOX2-dependent ROS production in the loss of PrxII from *mdx* muscle.

Peroxiredoxins are known to undergo irreversible hyperoxidation at conserved peroxidatic cysteine residues leading to inactivation^37,39^ and degradation via the 20S proteasome^40^. Therefore, we performed western blot analysis on WT, *mdx*, and *mdx*/p47^–/–^ muscle lysates using antibodies specific to hyperoxidized peroxiredoxin^41^. The level of hyperoxidized peroxiredoxin was significantly increased in *mdx* muscle compared to WT, and was fully restored to WT levels in *mdx*/p47^–/–^ muscle (Fig. 2e). While hyperoxidized PrxII can be reactivated by sulfiredoxin-catalyzed reduction^42^, we did not detect expression of sulfiredoxin in skeletal muscle (Supplementary Fig. 2b). Finally, *mdx*/p47^–/–^ muscle was significantly protected from ECC force loss compared to *mdx* muscle (Fig. 2f). Collectively, these data suggest that aberrant NOX2-dependent ROS signaling leads to PrxII hyperoxidation and degradation in *mdx* muscle.

### Myoglobin knockout protects *mdx* muscle from ECC force loss

**Fig. 3:**
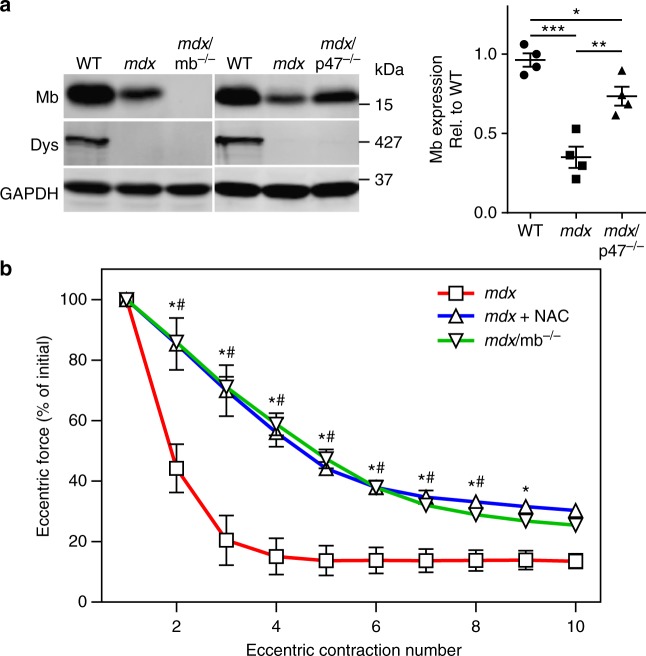
Genetic ablation of myoglobin partially protects *mdx* muscle from eccentric contraction-induced force loss. **a** Immunoblot analysis demonstrated the absence of myoglobin in *mdx*/mb^–/–^ muscle, that myoglobin levels are decreased in *mdx*, and that myoglobin levels are partially restored in *mdx*/p47^–/–^ gastrocnemius muscle; *n* = 4 for each genotype. *P < 0.05, **P < 0.01, ***P < 0.001; one-way ANOVA. **b** EDL muscles isolated from *mdx* and *mdx*/mb^–/–^ mice, or *mdx* muscles treated with 20 mM N-acetylcysteine (NAC) were subjected to 10 eccentric contractions and the forces measured expressed as a percentage of the force generated during the first eccentric contraction; *n* = 4 for WT and *mdx*+NAC; *n* = 9 for *mdx*/mb^–/–^; *mdx+NAC significantly different from *mdx* (*P* ≤ 0.05); ^#^*mdx*/mb^–/–^ significantly different from *mdx* (*P* ≤ 0.05); two-way ANOVA. Throughout, error bars represent means ± SEM

In skinned WT rodent myofibers, H_2_O_2_ can cause significant decrements in Ca^2+^-activated force when myoglobin is included in the bathing medium^43^. The combined effect is thought to be caused by hydroxyl radicals produced by the Fenton reaction of H_2_O_2_ with Fe^2+^ in myoglobin^44^. Given that *mdx* ECC force loss depends on an intact muscle fiber^21,24,45^, we investigated whether myoglobin mediates the inhibitory effect of ROS on the contractile function of *mdx* muscle fibers exposed to ECC. We crossed myoglobin knockout mice^46^ (mb^–/–^) onto the *mdx* background to generate *mdx*/mb^–/–^ mice. In verifying the absence of myoglobin in *mdx*/mb^–/–^ muscle by western blot analysis (Fig. 3a), we also showed that myoglobin levels are significantly decreased in *mdx* muscle and partially restored in *mdx*/p47^–/–^ muscle. Most importantly, knockout of myoglobin significantly protected *mdx* muscle from ECC force loss to the same extent as treatment of *mdx* muscle with the antioxidant *N*-acetylcysteine^47^ (Fig. 3b). These data suggest that myoglobin synergizes with ROS to effect ECC force loss in *mdx* muscle.

### PrxII ablation exacerbates ECC force loss in *mdx* muscle

**Fig. 4:**
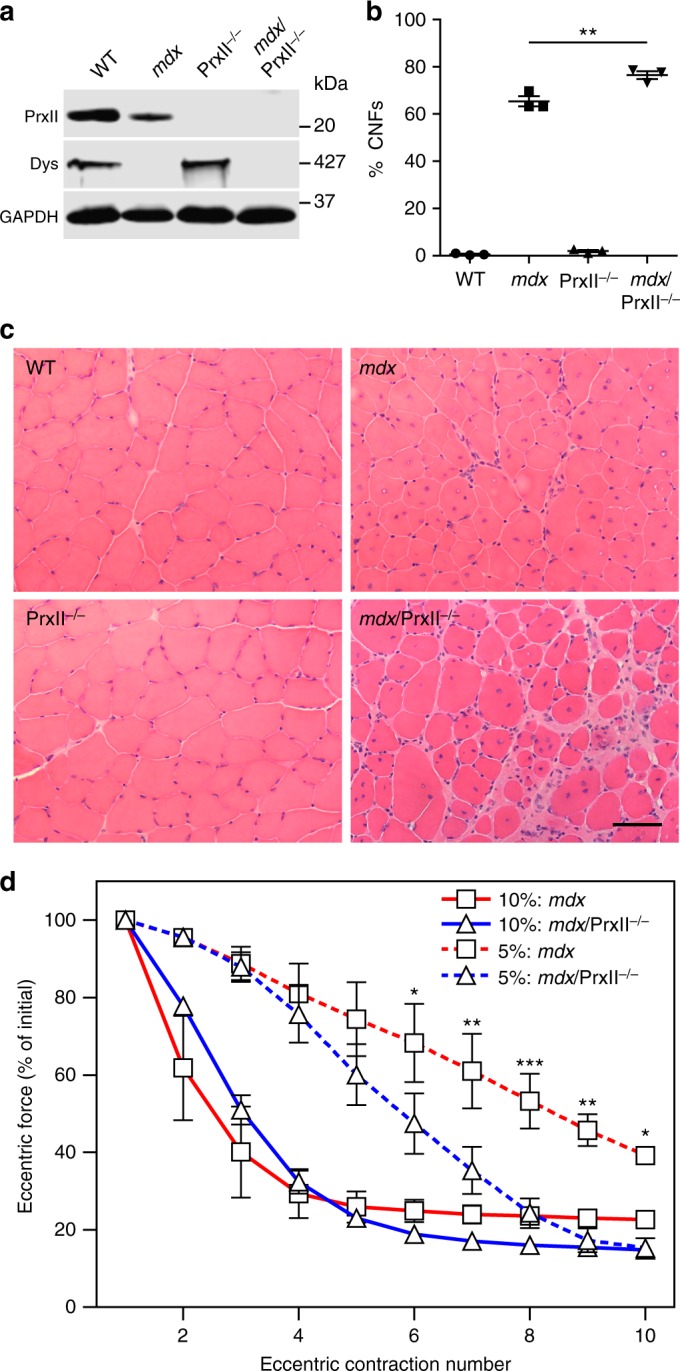
Genetic ablation of peroxiredoxin-2 further sensitizes *mdx* muscle to eccentric contraction-induced force loss. **a** Immunoblot analysis of PrxII in WT, *mdx*, PrxII^–/–^, and *mdx*/PrxII^–/–^ gastrocnemius demonstrated the absence of PrxII in PrxII^–/–^ and *mdx*/PrxII^–/–^ muscle. **b** A small but significant increase in the percentage of centrally nucleated fibers (%CNFs) was seen in *mdx*/PrxII^–/–^ versus *mdx* muscle quantified from 10 µm cryosections of TA stained with H&E. *n* = 3 for each genotype. **P < 0.01 for *mdx*/PrxII^–/–^ compared to *mdx*; one-way ANOVA. **c** Representative images of 10 µm cryosections of TA from WT, *mdx*, PrxII^–/–^, and *mdx*/PrxII^–/–^ stained with H&E. Scale bar: 50 µm. **d** EDL muscles isolated from *mdx* and *mdx*/PrxII^–/–^ mice were subjected to 10 eccentric contractions with either a 5% or 10% length change, and the forces measured expressed as a percentage of the force generated during the first eccentric contraction. There was no significant difference between *mdx* and *mdx*/PrxII^–/–^ with a 10% length change, but a 5% length change revealed a significant difference between *mdx* and *mdx*/PrxII^–/–^ for contractions 6–10; *n* = 4 for each genotype/condition. *P < 0.05, **P < 0.01, ***P < 0.001 compared to *mdx*; two-way ANOVA. Throughout, error bars represent means ± SEM

To further understand the role of PrxII in *mdx* ECC force loss, we crossed PrxII knockout mice^48^ (PrxII^–/–^) onto the *mdx* background to generate *mdx*/PrxII^–/–^ mice. Western blot analysis of PrxII^+/+^, PrxII^+/–^, and PrxII^–/–^ muscle lysates verified PrxII antibody specificity (Supplementary Fig. 3a) and confirmed the absence of PrxII in *mdx*/PrxII^–/–^ muscle (Fig. 4a). Hematoxylin and eosin (H&E) staining of muscle cryosections revealed that PrxII deletion resulted in a small, but significant exacerbation of histopathology in *mdx* muscle without an effect on WT muscle histology (Fig. 4b, c). In our standard ECC protocol that imposes a 10% change in muscle length to maximize force loss in *mdx* muscle, PrxII^–/–^ muscle was not susceptible to ECC force loss (Supplementary Fig. 3b), while ECC force loss in *mdx*/PrxII^–/–^ tracked to that measured in *mdx* (Fig. 4d). However, *mdx*/PrxII^–/–^ muscles showed significantly greater force loss than *mdx* muscle when exposed to a milder ECC protocol utilizing a 5% length change that elicits lower eccentric force (Fig. 4d). These data show that PrxII knockout increases the susceptibility of *mdx* muscle to ECC force loss.

### PrxII overexpression protects *mdx* muscle from ECC force loss

**Fig. 5:**
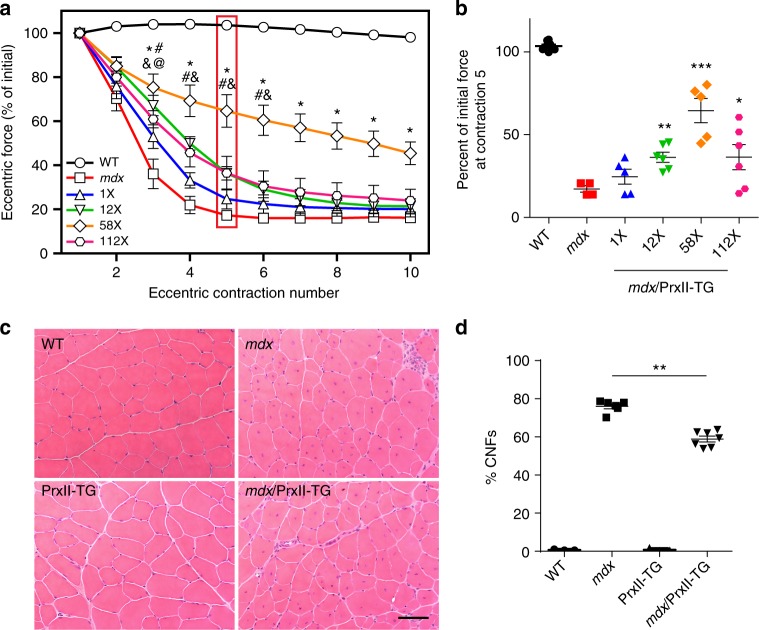
Muscle-specific peroxiredoxin-2 overexpression partially protects *mdx* muscle from eccentric contraction-induced force loss. **a** EDL muscles isolated from WT, *mdx*, and *mdx*/PrxII-TG lines expressing PrxII at 1-, 12-, 58-, and 112-fold relative to WT were subjected to 10 eccentric contractions and the forces measured expressed as a percentage of the force generated during the first eccentric contraction; *n* = 8 for WT; *n* = 4 for *mdx;* *n* = 5 for 1× and 58×; *n* = 6 for 12× and 112×. ^@^1× Significantly different from *mdx* (*P* < 0.05), ^#^12× significantly different from *mdx* (*P* < 0.05), *58× significantly different from *mdx* (*P* < 0.001), ^&^112× significantly different from *mdx* (*P* < 0.05); two-way ANOVA. **b** The force produced at contraction 5 for each line was presented as a percentage of initial force; *n* = same as in (**a**). *P < 0.05, **P < 0.01, ***P < 0.001 compared to *mdx*; one-way ANOVA. **c** Representative images of 10 µm cryosections of TA from WT, *mdx*, PrxII-TG, and *mdx*/PrxII-TG (58×) stained with H&E. Scale bar: 50 µm. **d** The 58-fold PrxII overexpression caused a small but significant decrease in the percentage of centrally nucleated fibers (%CNFs) in *mdx* TA muscle; *n* = 3 for WT and PrxII-TG; *n* = 6 for *mdx* *n;* = 7 for *mdx*/PrxII-TG. **P < 0.01 for *mdx*/PrxII-TG compared to *mdx*; one-way ANOVA. Throughout, error bars represent means ± SEM

Based on the detrimental effect of PrxII ablation on *mdx* muscle (Fig. 4), we tested the hypothesis that PrxII overexpression would protect *mdx* muscle from ECC force loss. We generated four lines of transgenic mice that overexpress PrxII specifically in skeletal muscle and crossed each line onto the *mdx* background to obtain four distinct lines of *mdx*/PrxII-TG mice. Quantitative western blot analysis demonstrated PrxII overexpression to 1×, 12×, 58× and 112× over WT levels in the four lines (Supplementary Fig. 4a,b). In our standard ECC protocol, we observed a clear dose-dependent protection of *mdx* muscle from ECC force loss with the greatest effect measured when PrxII was overexpressed 58× over WT (Fig. 5a, b). H&E staining of muscle cryosections from the 58× overexpressing *mdx*/PrxII-TG line revealed a small but significant decrease in the number of centrally nucleated fibers compared to *mdx* (Fig. 5c, d). Because restoration of PrxII to 1× WT levels did not protect *mdx* muscle from ECC force loss (Fig. 5a, b), the loss of PrxII from *mdx* muscle is not the primary cause of ECC force loss in *mdx* muscle, but rather likely a consequence of excessive ROS produced by NOX2 in response to mechanical activation.

### Protection of *mdx* muscle from ECC force loss by γ_cyto_-actin requires Cys272

**Fig. 6:**
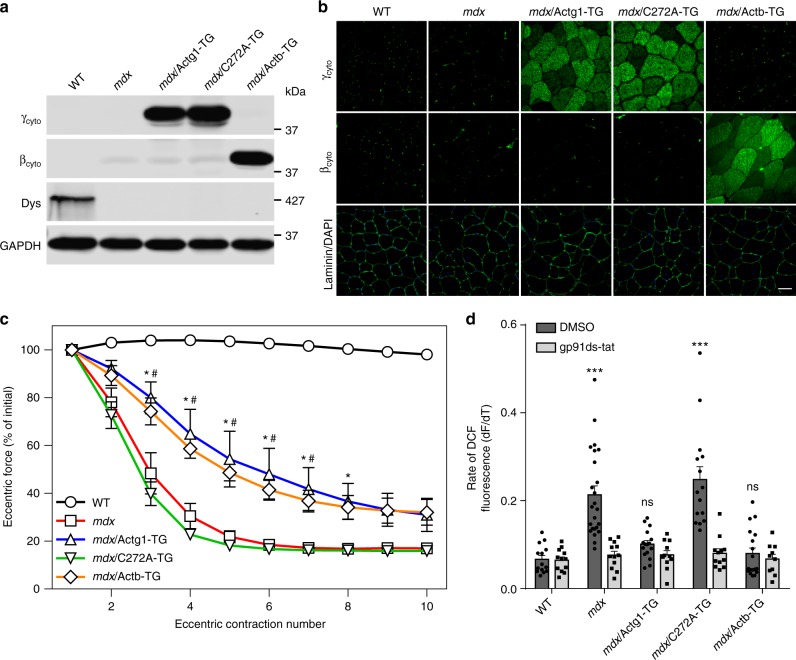
Cysteine 272 of γ_cyto_-actin is necessary for protection of *mdx* muscle from eccentric contraction-induced force loss. **a** Immunoblot comparison of γ_cyto_ (Actg1-TG), γ_cyto_^C272A^ (C272A-TG), and β_cyto_ (Actb-TG) overexpression in *mdx* gastrocnemius muscle. **b** Immunofluorescence analysis demonstrates similar distributions of γ_cyto_, γ_cyto_^C272A^, and β_cyto_ in 10 µm quadriceps cryosections. Scale bar = 50 µm. **c** EDL muscles isolated from WT, *mdx*, *mdx*/Actg1-TG, *mdx*/C272A-TG, and *mdx*/Actb-TG mice were subjected to 10 eccentric contractions and the forces measured expressed as a percentage of the force generated during the first eccentric contraction; *n* = 8 for WT and *mdx*/C272A-TG; *n* = 5 for *mdx*; *n* = 6 for *mdx*/Actg1-TG and *mdx*/Actb-TG. *The *mdx*/Actg1-TG significantly different from *mdx* (*P* ≤ 0.05), ^#^*mdx*/Actb-TG significantly different from *mdx* (*P* ≤ 0.05); two-way ANOVA. **d** Rate of DCF fluorescence in single flexor digitorum brevis (FDB) muscles from WT, *mdx*, *mdx*/Actg1-TG, *mdx*/C272A-TG, and *mdx*/Actb-TG mice exposed to cyclic stretch in the presence of DMSO (vehicle) or the NOX2 inhibitor gp91ds-tat; *n* = 9 for WT, *mdx*/Actg1-TG, and *mdx*/Actb-TG; *n* = 12 for *mdx*; *n* = 7 for *mdx*/C272A-TG. ***P < 0.001, ns no significance compared to WT; one-way ANOVA. Throughout, error bars represent means ± SEM

The protection of *mdx* muscle from ECC force loss by overexpression of γ_cyto_-actin, but not α_ca_-actin (Fig. 1c–h) led us to re-examine their highly homologous primary sequences. Interestingly, γ_cyto_-actin and β_cyto_-actin each contain 6 Cys residues while α_sk_-actin and α_ca_-actin only contain 5 Cys residues (Supplementary Fig. 5). The additional Cys unique to γ_cyto_-actin and β_cyto_-actin is located at position 272 (Supplementary Fig. 5, green highlight, blue circle) and has been shown to be the most reactive with H_2_O_2_^49^. To investigate the role of Cys272 within γ_cyto_-actin in protecting *mdx* muscle from ECC force loss, we generated transgenic mouse lines overexpressing the C272A mutant of γ_cyto_-actin (C272A-TG) as well as β_cyto_-actin (Actb-TG) specifically in skeletal muscle and crossed the lines onto the *mdx* background to identify those that best match γ_cyto_-actin overexpression in *mdx*/Actg1-TG (Fig. 6a). In addition, recombinant γ_cyto_-actin (for *mdx*/C272A-TG) and platelet actin (for *mdx*/Actb-TG) were used to generate standard curves for quantitative western blot analysis to verify equivalent expression of each actin transgene (Supplementary Fig. 6a-c). Uniformity of C272A and β_cyto_-actin overexpression was verified by immunofluorescence analysis (Fig. 6b), while H&E staining revealed no obvious change in the histopathology of *mdx* muscle associated with overexpression of C272A or β_cyto_-actin (Supplementary Fig. 6d). Most interestingly, β_cyto_-actin overexpression protected *mdx* muscle from ECC force loss to the same extent as γ_cyto_-actin, while ECC force loss in *mdx*/C272A-TG was not different from *mdx* (Fig. 6c). Finally, we measured the rate of NOX2-dependent ROS production in response to cyclic stretch of single myofibers^38^ isolated from WT, *mdx*, *mdx*/Actg1-TG, *mdx*/C272A-TG, and *mdx*/Actb-TG mice (Fig. 6d). Stretch-dependent, NOX2-mediated ROS signaling was significantly greater in *mdx* and *mdx*/C272A-TG compared to WT, *mdx*/Actg1-TG, or *mdx*/Actb-TG (Fig. 6d). These data demonstrate that γ_cyto_-actin and β_cyto_-actin overexpression protects *mdx* muscle from ECC force loss by blocking the stretch-dependent NOX2-mediated production of ROS and show that Cys272 is necessary for inhibition.

### Increased sarcolemmal damage in *mdx* muscle overexpressing PrxII following in vivo ECC

**Fig. 7:**
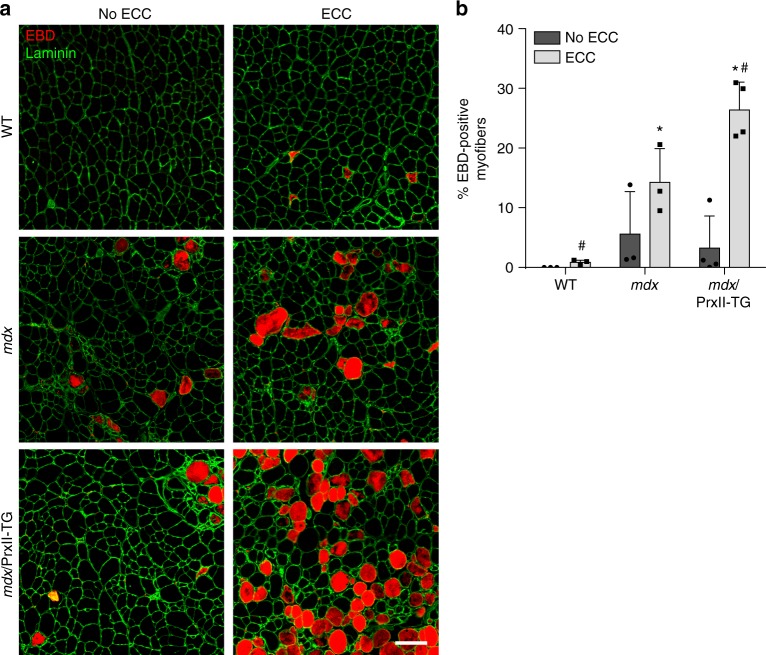
Muscle-specific peroxiredoxin-2 overexpression leads to increased sarcolemmal damage following long-term injurious eccentric contractions in *mdx* mice. **a** Fluorescent microscopy of WT, *mdx*, and *mdx*/PrxII-TG (58×) for Evans blue dye (EBD; red) and laminin (green). No ECC contralateral TA not subjected to eccentric contractions, ECC TA subjected to 70 eccentric contractions performed in vivo. Scale bar = 50 µm. **b** Quantification of the percentage of EBD-positive myofibers in WT, *mdx*, and *mdx*/PrxII-TG TA muscle either subjected to 70 eccentric contractions (ECCs) or not (no ECC); *n* = 3 for WT and *mdx;* *n* = 4 for *mdx*/PrxII-TG. *P < 0.05 compared to WT-ECC, ^#^*P* < 0.05 compared to *mdx*-ECC; one-way ANOVA

The rapid but mostly reversible inhibition of force production in *mdx* muscle exposed to ECC led us to consider that it may actually protect *mdx* muscle from damage analogous to an electrical circuit breaker. However, disabling the circuit breaker could lead to greater damage to *mdx* muscle exposed to ECC. To test this hypothesis, we measured sarcolemmal damage in WT, *mdx*, and 58X *mdx*/PrxII-TG following 70 eccentric contractions in vivo (Fig. 7a, b). While the unstressed contralateral muscle of *mdx*/PrxII-TG mice displayed similar levels of Evans blue dye (EBD) uptake compared to *mdx*, they presented with significantly more (27% vs. 14% in *mdx*) EBD-positive myofibers following 70 in vivo eccentric contractions (Fig. 7a, b). These data suggest that disabling ROS-mediated inhibition of force loss leads to greater injury following in vivo ECC in *mdx* muscle.

## Discussion

Our data best fit a model in which PrxII functions as an off-switch to regulate stretch-activated NOX2 signaling in normal skeletal muscle^50^, but is lost from *mdx* muscle through hyperoxidation and proteolytic degradation effected by aberrant NOX2-mediated ROS production. PrxII is known to suppress redox-mediated growth factor signaling^51–53^, while NOX2 in *mdx* skeletal muscle has been demonstrated to produce significantly more ROS^38^, particularly in response to mechanical stretch as occurs during ECC^27,29,30,54^. Importantly, treatment with the NOX2 inhibitor apocynin^27^, the non-specific antioxidant *N*-acetylcysteine^47^, or catalase overexpression^55^ has been shown to protect *mdx* muscle from ECC force loss, mirroring the effect we observed in *mdx*/Actg1-TG (Fig. 1e), *mdx*/p47^–/–^ (Fig. 2f), *mdx*/mb^–/–^ (Fig. 3b), *mdx*/PrxII-TG (Fig. 5a), and *mdx*/Actb-TG EDL muscles (Fig. 6c) (for more physiological parameters of all mouse lines, see Supplementary Table 1).

Our stretch-activated ROS experiments with single myofibers from transgenic models overexpressing γ_cyto_-actin, β_cyto_-actin, or the C272A mutant of γ_cyto_-actin (Fig. 6d) suggest that cytoplasmic actin overexpression directly inhibits NOX2 activity in *mdx* skeletal muscle. In vitro binding experiments and studies in nonmuscle cells have shown that actin can inhibit NOX2 activity by directly binding and sequestering the p40^phox^, p47^phox^, and/or p67^phox^ regulatory subunits^56,57^. Alternatively, the additional redox-sensitive Cys272 unique to γ_cyto_- and β_cyto_-actins^49,58^ may serve to shunt ROS-mediated oxidation away from conserved sulfhydryls necessary for contractile function. Future experiments will address both possibilities.

One poorly understood feature of ECC force loss in *mdx* muscle is its dependence on intact myofibers, because skinned fibers from *mdx* mice are no more susceptible to ECC than WT^21,24,45^. In studies of muscle fatigue, ROS in the form of H_2_O_2_ is thought to exert inhibitory effects on contractile proteins, yet extremely high concentrations of H_2_O_2_ are required to cause significant decrements in Ca^2+^-activated force loss in skinned myofibers^44,59^. However, the concentration of H_2_O_2_ required to elicit force loss is greatly reduced when myoglobin is included in the bathing medium^43,44^, which results in the production of highly reactive hydroxyl radicals through the reaction of H_2_O_2_ with Fe^2+^ in myoglobin^44^. Our experiments in *mdx*/mb^–/–^ mice (Fig. 3) suggest that myoglobin and H_2_O_2_ may catalyze similar Fenton chemistry in *mdx* muscle to cause ECC force loss with concomitant oxidative degradation of myoglobin.

While the ROS-based perturbations tested here by us and reported by others previously^27,47,55,60^ all demonstrated significant protection of *mdx* muscle from ECC force loss, the measured protection is incomplete. Other studies have implicated elevated cytosolic calcium^61–64^, loss of neuromuscular junction or sarcolemmal membrane excitability^19,20^, neuronal nitric oxide synthase^25^, and Akt/PKB signaling^24^ in *mdx* ECC force loss and our results are neither incompatible with nor mutually exclusive of such mechanisms. For example, NOX2 negatively regulates Akt activation in skeletal muscle exposed to oxidative stress^65^, while the activity of ion channels important for membrane excitability are sensitive to oxidation^66^. Although stretch-activated calcium channels are clearly one downstream effector of the ROS produced by NOX2^28^, it is not clear how an increase in cytosolic calcium could affect rapidly reversible ECC force loss as reported here (Fig. 1a, b) and elsewhere^67,68^. Perhaps the rapidly reversible component of ECC force loss, measured here over the course of 2 h, is due to reversible oxidation of one or more proteins regulating muscle contraction, while the slowly reversible component, or force loss that is recovered in the timeframe of several days^69^, is due to calcium-activated proteolysis of other muscle regulatory proteins. Alternatively, both the rapidly reversible and slowly reversible components of ECC force loss in *mdx* muscle could result from oxidative stress. In some pathological states, peroxiredoxins (including PrxII) are subject to over-oxidation such that reversibly sulfenylated cysteine residues become irreversibly sulfinylated or sulfonylated^53,70^. Such hyperoxidation may occur in proteins involved with contractility in *mdx* muscle, leading to their irreversible inactivation and contributing to the slowly reversible component of ECC force loss.

As noted in the Introduction, ECC force loss in *mdx* muscle is often referred to as “contraction-induced injury,” or “contraction-induced damage,” and muscle damage is operationally defined “as weakness which recovers very slowly after activity with a time course (4–8 days) similar to repair or regeneration”^18^. We measured rapid recovery of force in isolated *mdx* EDL muscles exposed to ECC (Fig. 1a). While our rapid recovery data are supported by other studies^67,68^, they are incompatible with the above definition of muscle damage. On the other hand, the rapidly reversible component of ECC force loss fits well with a reversible ROS-mediated inhibition of contractile force. This immediate and reversible inhibition of force in *mdx* may benefit the muscle in the long run, since more injurious contractions performed in vivo on *mdx* mice with decreased ROS signaling (*mdx*/PrxII-TG) led to increased sarcolemmal permeability (Fig. 7). Rather than serving as a readout for muscle damage, ours and others’ data collectively lead us to propose that ECC force loss may instead function as an adaptive circuit breaker that protects dystrophin-deficient muscle from potentially cell-lethal structural damage caused by continually repeated high force contractions.

## Methods

### Mice

All animals were housed and treated in accordance with the standards set by the University of Minnesota Institutional Animal Care and Use Committee. All animal experiments were approved by the University of Minnesota Institutional Animal Care and Use Committee under protocol numbers 1207A17501, 1506-32699A, and 1806-36018A. Mice were maintained on regular diet in a specific-pathogen-free facility on a 12 h light/dark cycle with continuous access to food and water. All wild-type mice used in this study were on the C57BL/10SnJ background. All transgenic overexpression mice are skeletal muscle-specific using the human skeletal actin (HSA) promoter. All mice on the *mdx* background utilized the C57BL/10ScSn-*Dmd*^*mdx*^/J strain of *mdx* mice from The Jackson Laboratory. Transgenic mice overexpressing γ_cyto_-actin on the *mdx* background (*mdx*/Actg1-TG) have been previously described^31^. Transgenic mice overexpressing α_cardiac_-actin (Coco) have been previously described^35^ and bred onto the *mdx* background in this study. Mice with a genetic deletion of the NOX2 scaffolding subunit p47^phox^ (p47^–/–^) were obtained from The Jackson Laboratory (B6(Cg)-*Ncf1*^*m1J*^/J) and bred onto the *mdx* background as previously described^38^. Mice with a genetic knockout of myoglobin (mb^–/–^) have been previously described^46^ and were bred onto the *mdx* background in this study. Mice lacking peroxiredoxin-2 (PrxII^–/–^) have been previously described^48^ and were rederived from sperm donated by Dr. Dae-Yeul Yu (Korean Research Institute of Bioscience and Biotechnology) and bred onto the *mdx* background in this study. Transgenic mice overexpressing peroxiredoxin-2 (PrxII-TG), γ_cyto_^C272A^ (C272A-TG), and β_cyto_-actin (Actb-TG) are described in this paper (Methods – Cloning and generation of transgenic mice), and all three were bred onto the *mdx* background, resulting in *mdx*/PrxII-TG, *mdx*/C272A-TG, and *mdx*/Actb-TG mice. For all transgenic lines on the *mdx* background, non-transgenic *mdx* littermates were used as controls. Animals used for physiological experiments were all 3 months of age, while mice used for all other experiments were 3–6 months of age. All mice used in this study were male.

### Antibodies and reagents

Primary antibodies used for western blotting were: mouse monoclonal anti-γ_cyto_-actin^71^ (1:1000; clone 2–4), mouse monoclonal anti-α_cardiac_-actin (1:1000; Sigma, A9357), rabbit polyclonal anti-PrxII (1:1,000; Sigma, R8656), mouse monoclonal anti-Dystrophin (1:100; Leica, NCL-DYS1), mouse monoclonal anti-Utrophin (1:100; Santa Cruz, sc-33700), mouse monoclonal anti-GAPDH (1:10,000; Sigma, G8795), rabbit polyclonal anti-GAPDH (1:10,000; Sigma, G9545), rabbit polyclonal anti-PrxI (1:500; Abcam, ab15571), mouse monoclonal anti-PrxIII (1:500; Abcam, ab16751), mouse monoclonal anti-PrxIV (1:500; Abcam, ab16943), mouse monoclonal anti-PrxV (1:500; Abcam, ab16944), rabbit polyclonal anti-PrxVI (1:1000; Sigma, P0058), rabbit polyclonal anti-PrxSO_3_ (1:200; Abcam, ab16830), rabbit polyclonal anti-Myoglobin (1:5000; Dako, A0324), mouse monoclonal anti-β_cyto_-actin (1:1000; Sigma, A1978), mouse monoclonal anti-gp91^phox^ (1:1000; BD Biosciences, 611414), mouse monoclonal anti-p67^phox^ (1:1000; BD Biosciences, 610912), rabbit polyclonal anti-p22^phox^ (1:200; Santa Cruz, sc-20781), mouse monoclonal anti-Rac1 (1:2000; Cytoskeleton, ARC03), rabbit polyclonal anti-p47^phox^ (1:1000; EMD Millipore, 07-500), rabbit polyclonal anti-p40^phox^ (1:500; EMD Millipore, 07-501), and rabbit polyclonal anti-Sulfiredoxin (1:1000; Proteintech, 14273-1-AP). Secondary antibodies used for western blotting were: DyLight 680 Goat anti-Mouse IgG (CST, 5470S), DyLight 680 Goat anti-Rabbit IgG (CST, 5366S), DyLight 800 Goat anti-Mouse IgG (CST, 5257S), and DyLight 800 Goat anti-Rabbit IgG (CST, 5151S) all at 1:10,000 dilutions.

Primary antibodies used for immunofluorescence were: rabbit polyclonal anti-γ_cyto_-actin^71^ (1:500; clone 7577), mouse monoclonal anti-α_cardiac_-actin (1:250; Sigma, A9357), rabbit polyclonal anti-Laminin (1:1000; Sigma, L9393), rat monoclonal anti-Laminin (1:1000; Sigma, L0663), mouse monoclonal anti-β_cyto_-actin-FITC (1:500; Abcam, ab6277), and rabbit polyclonal anti-Dystrophin^72^ (1:20; clone Rb2). Secondary antibodies used for immunofluorescence were: Alexa Fluor 488 Donkey anti-Mouse IgG (ThermoFisher, A-21202), Alexa Fluor 488 Donkey anti-Rabbit IgG (ThermoFisher, A-21206), Alexa Fluor 488 Goat anti-Rat IgG (ThermoFisher, A-11006), Alexa Fluor 568 Donkey anti-Mouse IgG (ThermoFisher, A-10037), Alexa Fluor 568 Goat anti-Rabbit IgG (ThermoFisher, A-11011), and Alexa Fluor 568 Goat anti-Rat IgG (ThermoFisher, A-11077) all at 1:500 dilutions.

Other reagents used were DH5α competent cells (Invitrogen, 18258012), DH10Bac competent cells (Invitrogen, 10361012), Sf9 insect cells (ATCC, CRL-1711), *N*-acetylcysteine (Sigma, A7250), DCFH-DA (6-Carboxy-2′,7′-Dichlorodihydrofluorescien Diacetate) (Invitrogen, C-400), gp91ds-tat (NOX2-specific peptide inhibitor) (Bio-Synthesis Inc., Lewisville, TX), FLAG peptide (University of Minnesota Genomics Center, Minneapolis, MN), anti-FLAG M2 affinity gel (Sigma, A2220), and human platelet actin protein (Cytoskeleton, APHL99).

### Ex vivo EDL force measurements

Contractile functions of EDL muscles were assessed according to methods described previously^73^. Mice were anesthetized with sodium pentobarbital (75–100 mg/kg body mass). EDL muscles were dissected and mounted on a 300B-LR dual-mode muscle lever system (Aurora Scientific Inc.) with 5–0 suture in a 1.2 mL bath assembly with oxygenated (95:5% O_2_/CO_2_) Krebs-Ringer bicarbonate (Krebs) buffer maintained at 25 °C. The stimulator and muscle lever system was controlled by computer using a KPCI-3108 interface board (Keithley Instruments) and TestPoint software (SuperLogics). Muscles were adjusted to their anatomical optimal length (*L*_o_) based on resting tension, with length being measured from the distal myotendonous junction to the proximal myotendonous junction using digital calipers. Prior to performing eccentric contractions, maximal isometric tetanic force (*P*_o_) was measured every 2 min by stimulating the muscle to contract for 200 ms at 175 Hz until force plateaued, with this value being designated “pre *P*_o_”, or simply “Pre”. In our standard ECC protocol, a series of 10 eccentric contractions were performed and the peak force of each contraction was recorded. For each ECC force measurement, the muscle was passively shortened to 95% *L*_o_ and then stimulated for 200 ms while the muscle was simultaneously lengthened to 105% *L*_o_ at a velocity of 0.5 *L*_o_/s. Each eccentric contraction was separated by 3 min of rest before performing the next eccentric contraction to prevent fatigue^74^. The force measured at each eccentric contraction was expressed as a percentage of the force produced during the first (“initial”) contraction. The value of “*n*” for all ECC experiments is defined as “number of mice”, and only one EDL per mouse was used for each experiment. This standard ECC protocol was used in Fig. 1e, h, Fig. 2f, Fig. 3b, Fig. 4d (10%: *mdx* and 10%: *mdx*/PrxII^–/–^), Fig. 5a, Fig. 6c, and Supplementary Fig. 3b. Experiments where variations of the standard ECC protocol were used are explained below.

For Fig. 1a, *mdx* muscles were subjected to either the standard ECC protocol described above or the same protocol substituting isometric contractions for eccentric contractions performed on the contralateral EDL. After each 10-contraction protocol, *P*_o_ was measured immediately (Post 0’) as well as at the 20, 40, 60, 90, and 120 min timepoints (Post 20’, Post 40’, etc.). Each *P*_o_ was then expressed as a percentage of the *P*_o_ measured before the 10 eccentric or isometric contractions (Pre). For the eccentric protocol, statistical significance was determined for the *P*_o_ measured at Post 20’ through Post 120’ compared to the *P*_o_ measured at Post 0’ (see Statistical analysis).

In Fig. 1b, *mdx* EDLs were subjected to only 5 eccentric contractions. The first muscle used a standard 3 min of rest between each eccentric contraction, while the contralateral muscle was allowed 30 min of rest between contractions.

Figure 3b involves a group where *N*-acetylcysteine (NAC) treatment was performed on *mdx* EDLs. For this group, NAC (Sigma-Aldrich) was dissolved directly into Krebs Buffer at 20 mM immediately prior to bath assembly^47^. The control *mdx* group used standard Krebs Buffer without NAC. For both groups, EDL muscles were incubated for 30 min so NAC could efficiently penetrate the muscle before being subjected to the standard ECC protocol.

For Fig. 4d, the standard ECC protocol was used for 10%: *mdx* and 10%: *mdx*/PrxII^–/–^ experiments (solid lines). For the other two experiments (dash lines), the standard ECC protocol was used except that a 5% length change was performed instead of a 10% change. Here, EDL muscles were passively shortened to 97.5% *L*_o_ and then stimulated for 200 ms while the muscle was simultaneously lengthened to 102.5% *L*_o_ at 0.25 *L*_o_/s, resulting in lower peak eccentric force being generated compared to the 10% length change.

### Immunoblot analysis

Gastrocnemius muscles from mice of the indicated genotypes were dissected, flash frozen in liquid N_2_, pulverized to a powder with a liquid N_2_-cooled mortar and pestle, and resuspended in 1% sodium dodecyl sulfate (SDS) in phosphate-buffered saline (PBS) with protease inhibitors (100 nM aprotinin, 1 mM benzamidine, 10 µM E-64, 10 µM leupeptin, 1 mM pepstatin A, 1 mM phenylmethanesulfonylfluoride, 1 µM calpain inhibitor I, and 1 µM calpeptin). Samples were then incubated at room temperature (RT) with end-over-end rotation for 30 min, followed by centrifugation at 20,817 × *g* for 10 min to clear lysates of insoluble material. Total protein concentration of the lysates was determined by A_280_ measurement (NanoDrop 1000, ThermoFisher Scientific). Lysates were then diluted to equal concentrations before Laemmli sample buffer was added and boiled for 5 min. Equal amounts of protein were then separated by SDS–polyacrylamide gel electrophoresis (SDS-PAGE), transferred to polyvinylidene difluoride (PVDF) membranes, blocked with 5% milk in PBS, and incubated with the indicated primary antibodies overnight at 4 °C. The next day, membranes were washed 4 × 5 min in 0.1% Tween in PBS before incubation with DyLight secondary antibodies (1:10,000 each) for 1 h at RT. Membranes were again washed 4 x 5 min before being imaged with an Odyssey Infrared Scanner (LI-COR Biosciences). Protein bands were then quantified using LI-COR Image Studio software.

### Muscle histology and immunofluorescence

TA muscles from each mouse line were cryopreserved in melting isopentane for 30 s and 10 µm transverse cryosections were obtained (Leica CM3050 S). For histology, sections were stained with H&E and imaged on a Leica DM5500 B microscope equipped with a Leica HC PLAN APO 20× objective. Centrally nucleated fibers (CNFs) were counted using the Cell Counter plugin on ImageJ software (NIH) and expressed as a percentage of the total number of myofibers (%CNFs). For immunofluorescence, sections were fixed in 4% paraformaldehyde in PBS at RT for 10 min and subsequently washed three times in PBS before being permeabilized in 0.1% Triton X-100 in PBS for 10 min at RT. Sections were then blocked in 5% bovine serum albumin (BSA) and 0.1% Triton X-100 in PBS for 1 h at RT before incubating with primary antibodies overnight at 4 °C. Slides were then washed three times in PBS before incubating with Alexa Fluor secondary antibodies (1:500 each) for 1 h at RT. Sections were finally wash three times in PBS and mounted in ProLong Gold Antifade with 4′,6-diamidino-2-phenylindole (DAPI) to visualize nuclei (ThermoFisher Scientific). Images were acquired on a Deltavision PersonalDV deconvolution microscope equipped with an Olympus UApo 20x objective.

### iTRAQ proteomics

TA muscles were dissected from 5 *mdx*/Actg1-TG mice and 3 non-transgenic *mdx* littermates and pulverized into powder with a liquid nitrogen-cooled mortar and pestle. We closely followed the protein extraction and preparation steps as well as offline peptide fractionation described previously^75^ with slight modifications. We added 10 µL of lysis buffer per milligram of tissue for protein extraction and sonicated. A 105 µL aliquot of each lysate was run in the Barocycler NEP2320 (Pressure Biosciences, South Easton, MA) after which a Bradford assay was performed to determine protein concentrations. We digested 100 µg of each sample with trypsin, performed solid-phase extraction (SPE) clean-up, and reacted 40 µg of each of the eight protein samples with one of the iTRAQ® 8-plex reagents (Sciex, Framingham, MA). iTRAQ labels 113, 114, and 115 were used to label peptides from the 3 *mdx* samples, while iTRAQ labels 116, 117, 118, 119, and 121 were used to label peptides from the 5 *mdx*/Actg1-TG samples. We mixed equal aliquots of each protein sample (40 µg), processed by SPE clean-up, and performed peptide fractionation by liquid chromatography (LC). We performed the second dimension capillary LC separation of peptides in-line with an Orbitrap Velos mass spectrometer (MS) as previously described^76^. Slight variations in the mass spectrometer acquisition method were: lock mass was not used, HCD activation time was 20 msec, dynamic exclusion duration was 15 s, and the minimum signal threshold for data dependent trigger was 20,000 counts.

We converted the raw MS datafiles to MGF files with MS Convert from ProteoWizard Toolkit^77^. We used ProteinPilot™ v4.5 (Sciex, Framingham, MA) for the database search and quantification report with the following parameters: National Center for Biotechnology Information (NCBI) RefSeq mouse (Taxonomy ID: 10088) protein database combined with the contaminants database (http://www.thegpm.org/cRAP/index.html); 8-plex peptide label sample type; cysteine methyl methanethiosulfonate; trypsin; instrument Orbi MS (1–3ppm) Orbi MS/MS; biological modifications ID focus; thorough search effort; detected protein threshold 0.05 (10%); competitive error margin 2.00; and false discovery rate (FDR) analysis invoked (with reversed database). FDR calculations were performed in ProteinPilot™ with the concatenated forward and reversed protein sequence databases according to the method previously reported^78^. ProteinPilot™ calculates an average protein relative fold change between two conditions along with a 95% confidence interval for the fold change and a *P* value for a test of the null hypothesis unity (1:1 ratio), which helps assess the statistical significance of a fold change.

It should be noted that we originally attempted to perform iTRAQ proteomics on 4 *mdx*/Actg1-TG mice versus 4 non-transgenic *mdx* littermates. After obtaining the high-confidence protein list results, we discovered that the sample labeled with iTRAQ 116, initially thought to be an *mdx* mouse, displayed significantly higher levels of γ_cyto_-actin, consistent with iTRAQ labels 117, 118, 119, and 121 (*mdx*/Actg1-TG mice). We then re-genotyped the set of mice used in the iTRAQ proteomic screen, which revealed that the mouse labeled with iTRAQ 116 was indeed *mdx*/Actg1-TG, and that this mouse had been mis-genotyped or mis-labeled, resulting in 5 *mdx*/Acg1-TG mice and 3 non-transgenic *mdx* littermates instead of 4 of each genotype. Uncovering this mistake increased our confidence in the iTRAQ experiment, as the data from the screen itself unveiled the error.

### Quantitative reverse transcription-polymerase chain reaction (qRT-PCR)

Gastrocnemius of WT, *mdx*, and *mdx*/Actg1-TG mice were flash frozen in liquid N_2_ and pulverized to a powder with a cooled mortar and pestle. Total RNA was then extracted from each sample using the Aurum Total RNA Mini Kit (Bio-Rad) following the manual’s instructions. RNA concentration and purity (A_260/280_ ratio) were determined using a NanoDrop 1000 spectrophotometer (ThermoFisher Scientific). Reverse transcription was performed using the iScript Advanced complementary DNA (cDNA) Synthesis Kit for qRT-PCR (Bio-Rad) using the same initial RNA amount (1 µg) for all samples. qPCR reactions were prepared with the SsoAdvanced Universal SYBR Green Supermix (Bio-Rad) and quantified on a CFX96 Real Time System C1000 Touch Thermal Cycler (Bio-Rad). Primer sets for both PrxII and HPRT (loading control) were generated with Primer-BLAST software (NIH) to amplify across an exon junction and are listed in Supplementary Table 2. Significance of gene expression differences was determined by one-way analysis of variance (ANOVA) with Tukey's post hoc test analysis. Resulting qRT-PCR reactions were further run on a 1% agarose gel for visualization.

### Protein expression and purification

FLAG-tagged PrxII and Fascin-1 proteins were expressed and purified in Sf9 insect cells using the Bac-to-Bac protocol (Invitrogen). Briefly, recombinant baculoviral DNA was transfected into a small culture of Sf9 insect cells using CellFectin II (Invitrogen). After 4 days, the media containing the recombinant baculovirus were harvested and the transfected cells were analyzed for protein expression by anti-FLAG western blot. Once expression was validated, large (250 mL) cultures were incubated for 3 days with amplified baculovirus before being harvested for protein purification. For purification, cells were lysed using 1% Triton X-100 in PBS (8 mM NaH_2_PO_4_, 42 mM Na_2_HPO_4_, 150 mM NaCl, pH 7.5) containing protease inhibitors (100 nM aprotinin, 1 mM benzamidine, 10 µM E-64, 10 µM leupeptin, 1 mM pepstatin A, and 1 mM phenylmethanesulfonylfluoride) and protein was purified using ANTI-FLAG M2 affinity gel (Sigma-Aldrich) as previously described^79–81^. Proteins were dialyzed into PBS overnight at 4 °C before being concentrated and used in in vitro F-actin and G-actin binding assays (Supplementary Fig. 1c-f). Recombinant γ_cyto_-actin was expressed in the Bac-to-Bac insect cell expression system and purified as previously described^82,83^ before being used as a standard curve in determination of C272A-TG and Actb-TG transgene concentrations (Supplementary Fig. 6a,b).

### F-actin cosedimentation assay

A previously described F-actin high speed cosedimentation assay^82^ was used to measure binding properties of PrxII and Fascin-1 (positive control). Briefly, human platelet actin (Cytoskeleton) was resuspended in G-Buffer (5 mM Tris-HCl pH 8.0, 0.2 mM CaCl_2_, 0.2 mM ATP, and 0.5 mM DTT), then induced to polymerize with the addition of 10× Polymerization Buffer (100 mM Tris pH 7.5, 500 mM KCl, 20 mM MgCl_2_, and 10 mM ATP). Recombinant FLAG-PrxII or FLAG-Fascin-1 (1 µM each) was then incubated with various concentrations of F-actin (0–15 µM) for 30 min at RT. Samples then underwent high speed centrifugation at 100,000 × *g* for 30 min at 4 °C. Resulting supernatant and pellet fractions were subjected to SDS-PAGE, stained with Coomassie blue, and scanned using the Licor Odyssey system allowing quantification of supernatant and pellet fractions. The values from these experiments were plotted in GraphPad Prism software and nonlinear regression analysis was performed.

### G-actin binding assay

Human platelet actin (Cytoskeleton) was resuspended in G-Buffer (5 mM Tris-HCl pH 8.0, 0.2 mM CaCl_2_, 0.2 mM ATP, and 0.5 mM DTT) to maintain actin in its globular form (G-actin). Then, 400 µL mixtures of 1 µM G-actin alone (negative control) and 1 µM G-actin + 1 µM FLAG-PrxII (experiment) were made. Then, 100 µL of both mixtures were taken as the “Load” fraction. Then, 100 µL of each protein mixture was added to 25 µL of ANTI-FLAG M2 affinity beads and incubated for 1 h at 4 °C with end-over-end rotation. The beads were then centrifuged at 106 × *g* for 3 min at 4 °C and the supernatant was collected as the “Void” fraction. The beads were then washed three times with 400 µL G-buffer before adding 100 µL of 0.1 mg/mL FLAG peptide in G-Buffer and incubating at RT for 10 min. Samples were then centrifuged as before and the supernatants were collected as the “Elute” fraction. To ensure the G-actin was indeed in its globular form, 100 µM of both protein mixtures was incubated at RT for 30 min before being subjected to high-speed centrifugation (100,000 x *g*) for 30 min at 4 °C. The supernatant and pellet fractions were then collected and run with each Load, Void, and Elute fraction on SDS-PAGE. Gels were then Coomassie stained and scanned on the Licor Odyssey imaging system.

### PrxII immunoprecipitation

PrxII was immunoprecipitated from *mdx*/Actg1-TG muscle using the Pierce Crosslink Magnetic IP/Co-IP Kit (ThermoFisher Scientific) following the manual’s instructions. Briefly, gastrocnemius muscles from *mdx*/Actg1-TG mice were flash frozen and pulverized to a powder with a cooled mortar and pestle. *mdx*/Actg1-TG muscles were used because γ_cyto_-actin is expressed at such low levels in WT mice that it cannot be detected with typical western blotting. Samples were then solubilized with the kit’s IP Lysis Buffer (25 mM Tris pH 7.4, 150 mM NaCl, 1 mM EDTA, 1% NP-40, and 5% glycerol) supplemented with protease inhibitors (100 nM aprotinin, 1 mM benzamidine, 10 µM E-64, 10 µM leupeptin, 1 mM pepstatin A, 1 mM phenylmethanesulfonylfluoride, 1 µM calpain inhibitor I, and 1 µM calpeptin) by incubating for 30 min at 4 °C with end-over-end rotation. Lysates were then cleared by centrifugation at 20,817 × *g* for 10 min at 4 °C and protein concentrations were determined using the Pierce BCA Protein Assay Kit (ThermoFisher Scientific). Samples were diluted to 0.5 mg/mL in 1 mL of IP Lysis Buffer. During the protein sample preparation, 10 µg PrxII antibody (Sigma) was coupled to Protein A/G magnetic beads and crosslinked using DSS (disuccinimidyl suberate). Diluted protein was then incubated with the crosslinked magnetic beads for 1 h at RT. Beads were washed thoroughly before the sample was eluted with low pH Elution Buffer. Load, Void, Wash, and Elute fractions collected during the immunoprecipitation were subjected to SDS-PAGE, transferred to PVDF membranes, blocked with 5% milk in PBS, and probed with PrxII (Sigma) and γ_cyto_-actin (clone 2–4) primary antibodies overnight at 4 °C. Membranes were incubated with DyLight secondary antibodies before being scanned on the Licor Odyssey imaging system.

### Cloning and generation of transgenic mice

Human peroxiredoxin-2 plasmid cDNA was purchased from DNASU Plasmid Repository (Cat #HsCD00076134), PCR amplified, inserted into the Gateway entry vector pENTR/D-TOPO (Invitrogen), and sequence verified. All PCRs were performed using PfuUltra Fusion HS DNA Polymerase (Agilent Technologies). To make recombinant FLAG-PrxII protein, pENTR/D-TOPO-PRDX2 was N-terminally FLAG-tagged (pENTR/D-TOPO-N-FLAG-PRDX2) via PCR using primers that amplified the entire plasmid with FLAG sequence overhangs. The linear PCR product was circularized via the addition of T4 polynucleotide kinase and T4 DNA ligase (New England Biolabs) and sequence verified. Once verified, the entry vector was recombined into the Gateway insect cell destination vector pDEST8 (pDEST8-N-FLAG-PRDX2) using LR Clonase II (Invitrogen) and subsequently expressed in Sf9 insect cells using the Bac-to-Bac system (Invitrogen; see Protein expression and purification). To generate the PrxII-TG transgene, pENTR/D-TOPO-PRDX2 was recombined into a pDEST8 destination vector already containing the HSA promoter followed by the VP1 intron and tandem SV40 polyadenylation sequences (pDEST8-HSA-VP1-SV40-SV40). The PrxII cDNA was inserted in-frame between the VP1 intron and the first SV40 polyadenylation sequence (pDEST8-HSA-VP1-PRDX2-SV40-SV40). The construct was transformed into DH5α bacteria cells and extracted using the Wizard *Plus* SV Minipreps DNA Purification System (Promega). The DNA fragment from the HSA promoter through the polyadenylation sequence was restriction digested to linearize the transgene, gel purified via the QIAEX II Gel Extraction Kit (Qiagen), and sent to the Murine Genetics Core at The Scripps Research Institute for pronuclear microinjection into fertilized C57BL/6 zygotes, which were then implanted into pseudo-pregnant female mice.

We obtained Fascin-1 cDNA (FSCN1) as a kind gift from Dr. Steven Almo of the Albert Einstein College of Medicine. FSCN1 was PCR amplified, inserted into the Gateway entry vector pENTR/D-TOPO (Invitrogen), and sequence verified. To make recombinant FLAG-Fascin-1 protein, pENTR/D-TOPO-FSCN1 was N-terminally FLAG-tagged (pENTR/D-TOPO-N-FLAG-FSCN1) via PCR using primers that amplified the entire plasmid with FLAG sequence overhangs. The linear PCR product was circularized via the addition of T4 polynucleotide kinase and T4 DNA ligase (New England Biolabs) and sequence verified. Once verified, the entry vector was recombined into the Gateway insect cell destination vector pDEST8 (pDEST8-N-FLAG-FSCN1) using LR Clonase II (Invitrogen) and subsequently expressed in Sf9 insect cells using the Bac-to-Bac system (Invitrogen; see Methods - Protein expression and purification).

To generate C272A-TG and Actb-TG transgenes, both the pDEST8-HSA-VP1-C272A-SV40-SV40 (C272A-TG) and pDEST8-HSA-VP1-ACTB-SV40-SV40 (Actb-TG) constructs were cloned based on the original pDEST8-HSA-VP1-ACTG1-SV40-SV40 (Actg1-TG) construct previously described^34^. The C272A-TG construct was generated via site-directed mutagenesis using the QuikChange II XL kit (Agilent Technologies, Cat. no. 200521) according to the manufacture protocols and sequence verified. Primers were designed to allow a two-nucleotide change within the codon normally encoding Cysteine to then encode for Alanine at position 272 (TGT to GCT). For Actb-TG, only the ACTG1 nucleotides that coded for the 4 γ_cyto_-actin-specific amino acids (Glu2, Glu3, Glu4, and Ile10) were altered to instead code for the 4 β_cyto_-actin-specific amino acids (Asp2, Asp3, Asp4, and Val10) by editing the wobble base in each codon within the construct. This ensures any differences described between transgenic mice are only due to the altered amino acids, and not plasmid differences. The Actb-TG construct was generated via PCR using primers designed such that they amplified the Actg1-TG plasmid with ACTB-specific overhangs. The linear PCR product was circularized via the addition of T4 polynucleotide kinase and T4 DNA ligase (New England Biolabs) and sequence verified. Both constructs were transformed into DH5α bacteria cells and extracted using the Wizard *Plus* SV Minipreps DNA Purification System (Promega). The DNA fragment from the HSA promoter through the polyadenylation sequence was restriction digested to linearize the transgene, gel purified via the QIAEX II Gel Extraction Kit (Qiagen), and sent to the Murine Genetics Core at The Scripps Research Institute for pronuclear microinjection into fertilized C57BL/6 zygotes, which were then implanted into pseudo-pregnant female mice.

Resultant PrxII-TG, C272A-TG, and Actb-TG transgenic founder mice were identified by PCR using HSA-specific primers (Supplementary Table 2) and crossed with C57BL/10 mice to check for transgene transmission. Transgenic PrxII (PrxII-TG), γ_cyto_-actin (C272A-TG), and β_cyto_-actin (Actb-TG) protein expression was assessed in several different muscles from each transgenic line crossed onto the *mdx* (C57BL/10ScSn-*Dmd*^*mdx*^/J) background using quantitative western blotting. All *mdx*-transgenic mice used in this study were compared with non-transgenic littermate *mdx* mice as controls.

### Stretch-induced ROS measurements

Flexor digitorum brevus (FDB) muscles were surgically isolated and incubated in Dulbecco's modified Eagle's medium (DMEM; ThermoFisher Scientific) containing 0.1% penicillin–streptomycin (ThermoFisher Scientific) and 0.4% Collagenase A (Sigma-Aldrich) at 37 °C for 2 h. Single FDB fibers were isolated by gentle trituration in DMEM containing 0.1% penicillin–streptomycin and 10% fetal bovine serum (ThermoFisher Scientific) and incubated at 37 °C, 5% CO_2_ until used, typically 16–20 h later. Isolated FDB fibers were washed three times with HEPES solution (120 mM NaCl, 4.7 mM KCl, 1.8 mM CaCl_2_, 600 µM MgSO_4_, 1.6 mM NaHCO_3_, 130 µM NaH_2_PO_4_, 7.8 mM Glucose, and 20 mM HEPES) and loaded with 15 µM DCFH-DA (Invitrogen) for 25 min at RT in the dark. The fibers were then washed three times with HEPES solution containing either dimethyl sulfoxide (DMSO; 0.1%) or gp91ds-tat (10 µM) and the DCFH-DA dye was allowed to de-esterify for 15 min at RT in the dark. The ends of each fiber were attached to a micro-glass pipet coated with a biological adhesive (ECM Gel from Engelbreth-Holm-Swarm murine sarcoma, Sigma-Aldrich) and connected to micro-manipulators (Sutter Instruments). DCF Fluorescence (Ex: 480 nm, Em: 535/40 nm) and sarcomere length were acquired using an IonOptix system (Westwood, MA) atop a Motic AE31 microscope equipped with a 40× objective (Olympus UAP040X3/340). Each fiber was cyclically stretched to 110% of resting sarcomeric length (2–2.2 µm) at 10 µm/s for 15 min. The rate of DCF fluorescence was reported during the last 2.5 min of stretch.

### Evans blue dye assay

EBD was diluted in PBS to a final concentration of 5 mg/mL and was filter-sterilized with a 0.2 µm filter. EBD was administered by intraperitoneal injection (100 µL of diluted EBD per 10 g body weight) 24 h before the mice were subjected to eccentric contractions performed in vivo as previously described^84^. Briefly, the left hind limb was depilated and aseptically prepared and the foot was placed in a foot plate attached to a servomotor (Model 300B‐LR; Aurora Scientific, Aurora, Ontario, Canada) and Pt–Ir electrode wires (Model E2‐12; Grass Technologies, West Warwick, RI, USA) were inserted percutaneously on either side of the peroneal nerve. Contractions were induced via stimulation of the peroneal nerve at a frequency of 150 Hz by a stimulator and stimulus isolation unit (Models S48 and SIU5, respectively; Grass Technologies). Anterior crural muscles were injured by performing 70 electrically stimulated eccentric contractions (each contraction separated by 10 s), during which the foot was passively rotated from 0° to 19° dorsiflexion followed by 38° of plantarflexion at 2000°/s using the optimized voltage. Both the TA subjected to ECC and the contralateral TA (control) of each mouse were dissected and cryopreserved in melting isopentane before obtaining 10 µm cryosections at the mid-belly of each muscle. Sections were fixed in −20 °C acetone for 5 min, washed in PBS, blocked for 30 min at room temperature with 5% BSA/PBS, and counterstained with laminin (1:500; Sigma-Aldrich L9393) for 2 h at RT. Sections were then washed in PBS and incubated with anti-Rabbit Alexa Flour 488 (1:500; ThermoFisher Scientific) for 1 h at RT. Sections were washed a final time in PBS and mounted in ProLong Golf Antifade with DAPI (ThermoFisher Scientific). Images were acquired on a Leica DM5500 B microscope equipped with a Leica HC PLAN APO 10× objective and stitched together with LASX software (Leica) to allow visualization of the entire TA. MyoVision software (https://www.uky.edu/chs/muscle/myovision) was used to determine the percentage of EBD-positive myofibers in whole-TA images^85^.

### Statistical analysis

All statistics were calculated using GraphPad Prism software. All data are presented as mean ± SEM in force loss graphs, dot plots, and bar graphs. In force loss graphs, two-way ANOVA with Bonferroni post hoc test analyses were performed. For dot plots and bar graphs, one-way ANOVA with Tukey's post hoc test analyses were performed. For applicable experiments, the exact value of *n* (defined by number of animals) and the definition of significance can be found in the figure legend.

## Electronic supplementary material

Supplementary Information

Supplementary Data 1

Supplementary Data 2

Description of Additional Supplementary Files

## Data Availability

The data that support the findings of this study are available from the corresponding author upon reasonable request. The mass spectrometry proteomics data have been deposited to the ProteomeXchange Consortium via the PRIDE partner repository with the dataset identifier PXD009680. A reporting summary for this article is available as a Supplementary Information file.
