# Supplementary material for: Loss of peroxiredoxin-2 exacerbates eccentric contraction-induced force loss in dystrophin-deficient muscle
Source: Nat Commun. 2018 Nov 30;9:5104. doi: 10.1038/s41467-018-07639-3 (PMC6269445; doi:10.1038/s41467-018-07639-3)
Supplement: Supplementary file 1 — Supplementary Information [file 41467_2018_7639_MOESM1_ESM.pdf]

Loss of peroxiredoxin-2 exacerbates eccentric contraction-induced force loss in dystrophin-deficient muscle

Olthoff et al.

Supplementary Information

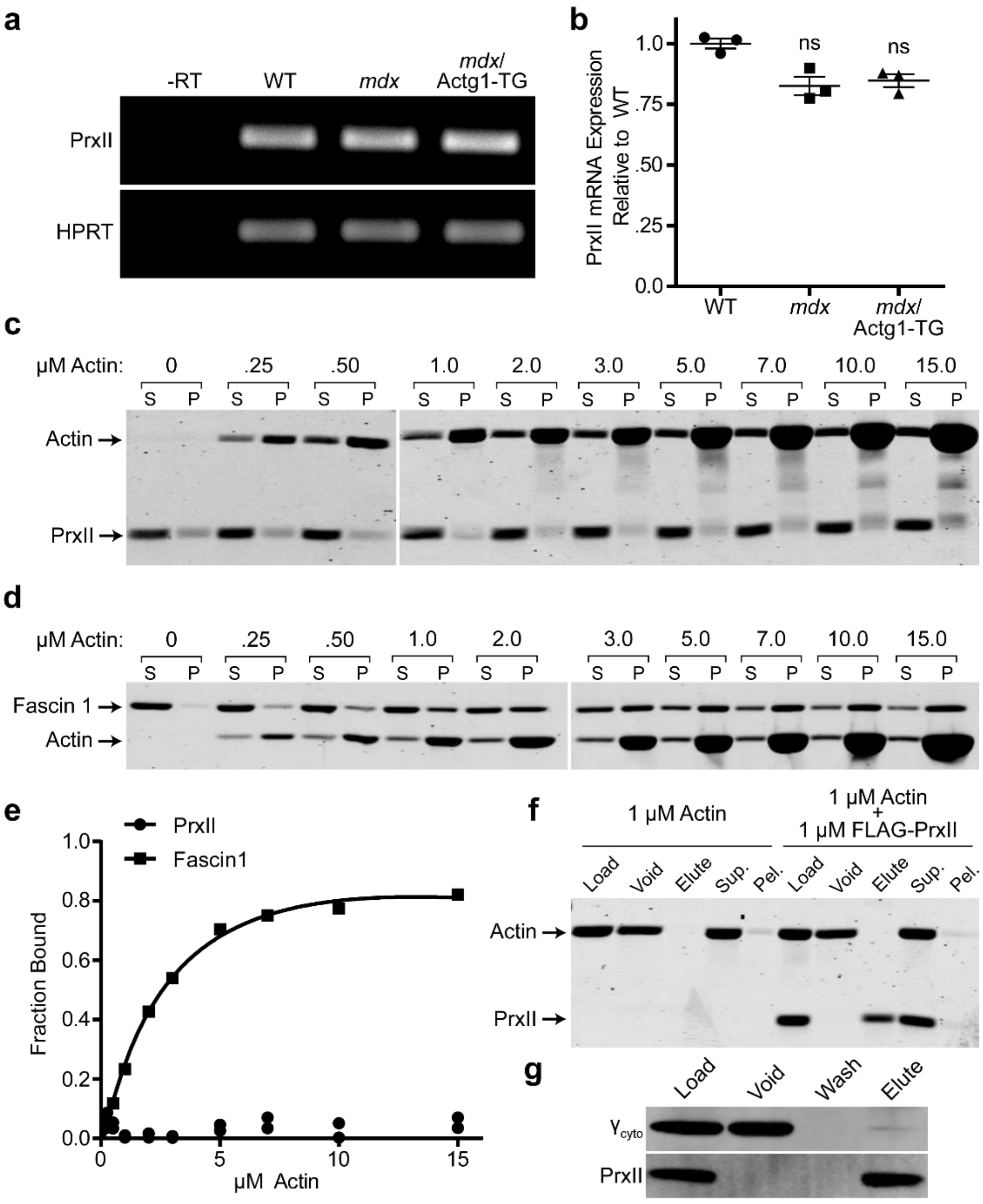

Supplementary Figure 1 Decreased peroxiredoxin-2 in *mdx* muscle is post-transcriptional and recovery of protein levels in *mdx*/Actg1-TG is independent of direct γ_{cyto}-actin binding. (**a,b**) qRT-PCR analysis of PrxII transcript in WT, *mdx*, and *mdx*/Actg1-TG gastrocnemius muscles. n = 3 per genotype. ns = no significance; one-way ANOVA. Error bars represent mean ± SEM. (**c**) F-actin cosedimentation assay with increasing platelet actin concentrations (0 – 15 µM) and constant concentrations of recombinant PrxII (1 µM). (**d**) F-actin cosedimentation assay with increasing platelet actin concentrations (0 – 15 µM) and constant concentrations of recombinant Fascin-1 (1 µM). (**e**) Binding curves of PrxII and Fascin-1 with platelet actin fitted with regression analysis. (**f**) G-actin binding assay with either 1 µM platelet actin only, or 1 µM platelet actin + 1 µM FLAG-PrxII. (**g**) Immunoprecipitation of PrxII from *mdx*/Actg1-TG gastrocnemius muscle immunoblotted for both γ_{cyto}-actin and PrxII.

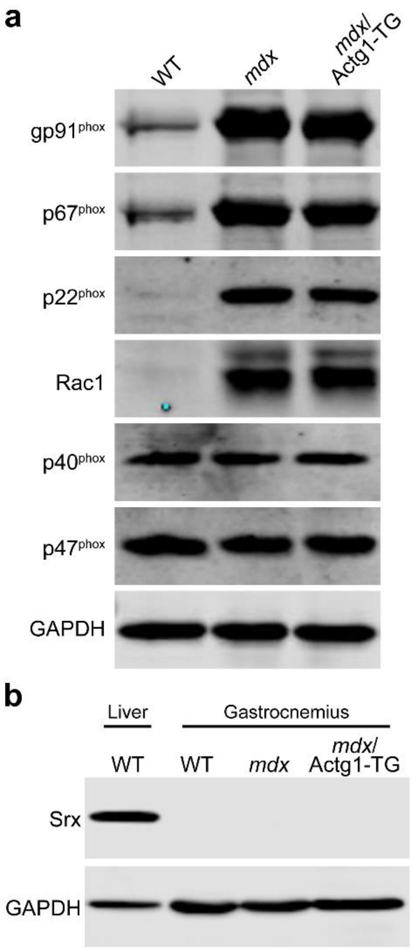

Supplementary Figure 2 Validation of NOX2 increase in *mdx* muscle and lack of sulfiredoxin in skeletal muscle. (**a**) Immunoblot analysis of all six NOX2 subunits in WT, *mdx*, and *mdx*/Actg1-TG gastrocnemius muscles. (**b**) Immunoblot analysis of sulfiredoxin in WT liver (positive control), as well as WT, *mdx*, and *mdx*/Actg1-TG gastrocnemius.

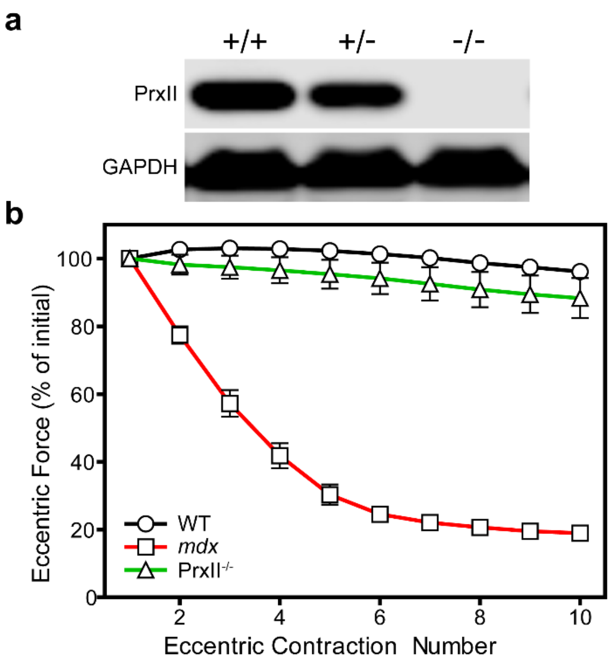

Supplementary Figure 3 Validation of PrxII-specific antibody and lack of effect of PrxII deletion of ECC-induced force loss in WT muscle. (**a**) Immunoblot analysis of PrxII (Sigma-Aldrich, R8656) in PrxII$^{+/+}$, PrxII$^{+/-}$, and PrxII$^{-/-}$ gastrocnemius. (**b**) EDL muscles isolated from WT, *mdx*, and PrxII$^{-/-}$ mice were subjected to 10 eccentric contractions and the force measured at each contraction expressed as a percentage of the force produced during the first contraction. n = 3 for all genotypes. Error bars represent mean ± SEM.

a

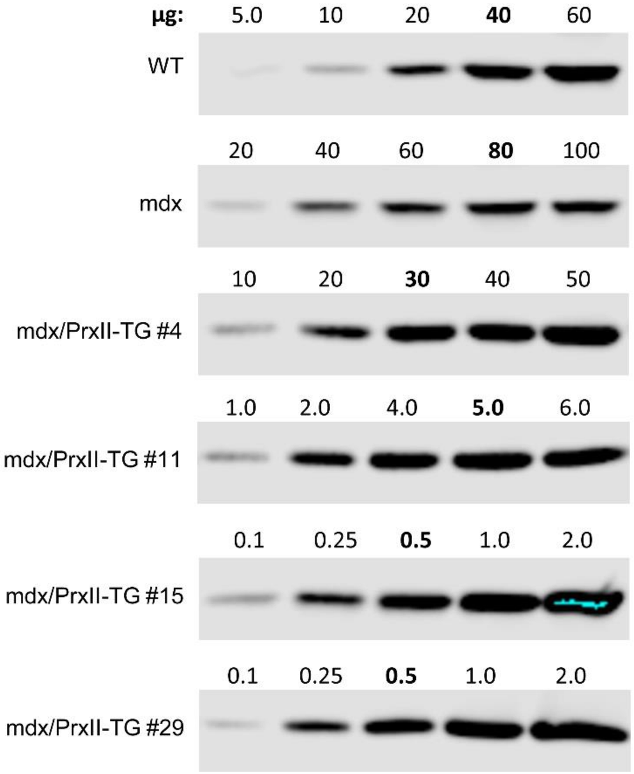

b

PrxII-TG Line	Overexpression Rel. to WT
#4	1.33 ±0.22
#11	11.93 ±1.49
#15	58.32 ±10.31
#29	111.70 ±9.84

Supplementary Figure 4 Relative quantitation of PrxII overexpression levels in *mdx* muscle. (**a**) Linear ranges of PrxII immunoreactivity were found for WT, *mdx*, and each *mdx*/PrxII-TG line (#4, #11, #15, and #29). The amount of lysate (in µg) in each line that best matched the signal seen in 40 µg of WT lysate is labeled in bold. (**b**) Using the bold values in (A) for each *mdx*/PrxII-TG line compared to 40 µg of WT, PrxII overexpression levels relative to WT were obtained. n ≥ 3 for each *mdx*/PrxII-TG line.

alpha-skeletal actin

```
Ac-DEDETTALVCDNGSGLVKAGFAGDDAPRAVFPSIVGRPRHQGVMVGMGQKDSYVGDEAQSKRGILTL
KYPIEHGIITNWDDMEKIWHHTFYNELRVAPEEHPTLLTEAPLNPKANREKMTQIMFETFNVPAMYVAIQ
AVLSLYASGRTTGIVLDSGDGVTHNVPIYEGYALPHAIMRLDLAGRDLTDYLMKILTERGYSFVTTAERE
IVRDIKEKLCYVALDFENEMATAASSSSLEKSYELPDGQVITIGNERFRCPETLFQPSFIGMESAGIHET
TYNSIMKCDIDIRKDLYANNVMSGGTTMYPGIADRMQKEITALAPSTMKIKIIAPPERKYSVWIGGSILA
SLSTFQQMWITKQEYDEAGPSIVHRKCF
```

alpha-cardiac actin

```
Ac-DDEETTALVCDNGSGLVKAGFAGDDAPRAVFPSIVGRPRHQGVMVGMGQKDSYVGDEAQSKRGILTL
KYPIEHGIITNWDDMEKIWHHTFYNELRVAPEEHPTLLTEAPLNPKANREKMTQIMFETFNVPAMYVAIQ
AVLSLYASGRTTGIVLDSGDGVTHNVPIYEGYALPHAIMRLDLAGRDLTDYLMKILTERGYSFVTTAERE
IVRDIKEKLCYVALDFENEMATAASSSSLEKSYELPDGQVITIGNERFRCPETLFQPSFIGMESAGIHET
TYNSIMKCDIDIRKDLYANNVLSGGTTMYPGIADRMQKEITALAPSTMKIKIIAPPERKYSVWIGGSILA
SLSTFQQMWISKQEYDEAGPSIVHRKCF
```

gamma-cytoplasmic actin

```
Ac-EEEIAALVIDNGSGMCKAGFAGDDAPRAVFPSIVGRPRHQGVMVGMGQKDSYVGDEAQSKRGILTLK
YPIEHGIVTNWDDMEKIWHHTFYNELRVAPEEHPVLLTEAPLNPKANREKMTQIMFETFNTPAMYVAIQA
VLSLYASGRTTGIVMDSGDGVTHTVPIYEGYALPHAILRLDLAGRDLTDYLMKILTERGYSFTTTAEREI
VRDIKEKLCYVALDFEQEMATAASSSSLEKSYELPDGQVITIGNERFRCPEALFQPSFLGMESCGIHETT
FNSIMKCDVDIRKDLYANTVLSGGTTMYPGIADRMQKEITALAPSTMKIKIIAPPERKYSVWIGGSILAS
LSTFQQMWISKQEYDESGPSIVHRKCF
```

beta-cytoplasmic actin

```
Ac-DDDIAALVVDNGSGMCKAGFAGDDAPRAVFPSIVGRPRHQGVMVGMGQKDSYVGDEAQSKRGILTLK
YPIEHGIVTNWDDMEKIWHHTFYNELRVAPEEHPVLLTEAPLNPKANREKMTQIMFETFNTPAMYVAIQA
VLSLYASGRTTGIVMDSGDGVTHTVPIYEGYALPHAILRLDLAGRDLTDYLMKILTERGYSFTTTAEREI
VRDIKEKLCYVALDFEQEMATAASSSSLEKSYELPDGQVITIGNERFRCPEALFQPSFLGMESCGIHETT
FNSIMKCDVDIRKDLYANTVLSGGTTMYPGIADRMQKEITALAPSTMKIKIIAPPERKYSVWIGGSILAS
LSTFQQMWISKQEYDESGPSIVHRKCF
```

= Conserved

= Muscle actin only

= Cytoplasmic actin only

= Oxidation known

= Oxidation known in cytoplasmic actin only

Supplementary Figure 5 Cysteine residues of striated muscle ($\alpha_{skeletal}$ and $\alpha_{cardiac}$) and cytoplasmic (γ_{cyto} and β_{cyto}) actins. Amino acid sequences of $\alpha_{skeletal}$-, $\alpha_{cardiac}$-, γ_{cyto}-, and β_{cyto}-actin are listed. Cysteines conserved in all four actins are highlighted in yellow, while cysteines present in muscle actins only are highlighted in red and cysteines present in only cytoplasmic actins are highlighted in green. Cysteines that are known from the literature to undergo oxidation *in vivo* are underlined in blue, while oxidative cysteines unique to cytoplasmic actins are circled in blue.

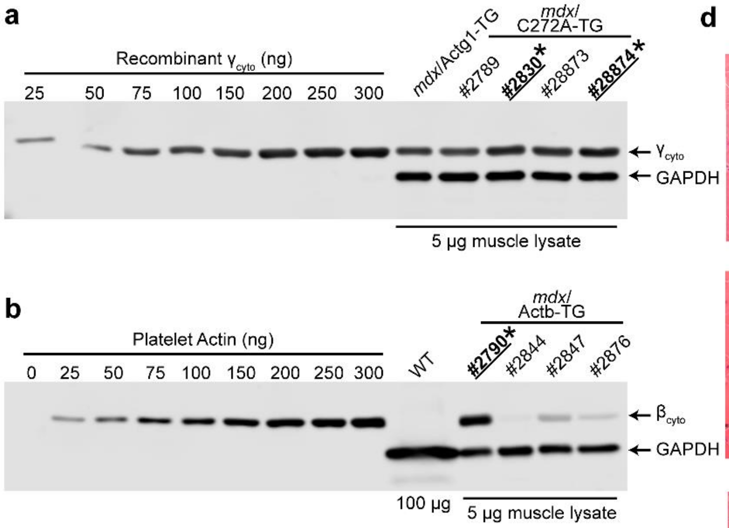

c

Transgene	ng per 1 µg lysate
mdx/Actg1-TG	18.18
mdx/C272A-TG #2830	29.38
mdx/C272A-TG #28874	33.03
mdx/Actb-TG #2790	30.55

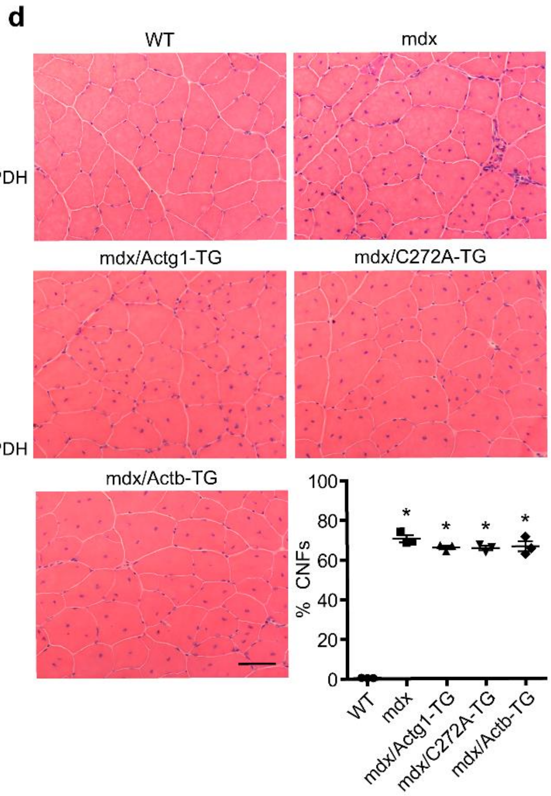

Supplementary Figure 6 Determination of γ_{cyto}^{C272A} and β_{cyto} protein concentrations and histopathology in *mdx*/C272A-TG and *mdx*/Actb-TG muscle. (**a**) Determination of γ_{cyto}-actin protein expression in 4 *mdx*/C272A-TG lines of mice compared to *mdx*/Actg1-TG using a standard curve of recombinant γ_{cyto}-actin. The standard curve and muscle lysates were immunoblotted for γ_{cyto}-actin. (**b**) Determination of β_{cyto}-actin protein expression in 4 *mdx*/Actb-TG lines of mice using a standard curve of platelet actin. The standard curve and muscle lysates were immunoblotted for β_{cyto}-actin. (**c**) Concentrations of γ_{cyto}-actin in the two surviving *mdx*/C272A-TG lines (#2830 and #28874) and β_{cyto}-actin in the one surviving *mdx*/Actb-TG line (#2790) were calculated along with γ_{cyto}-actin in *mdx*/Actg1-TG mice. (**d**) Representative H&E staining and percentage of centrally nucleated fibers (%CNFs) in 10 µm cryosections of quadriceps from WT, *mdx*, *mdx*/Actg1-TG, *mdx*/C272A-TG, and *mdx*/Actb-TG. n = 3 per genotype. *$P < 0.001$ compared to WT; one-way ANOVA. Error bars represent mean ± SEM.

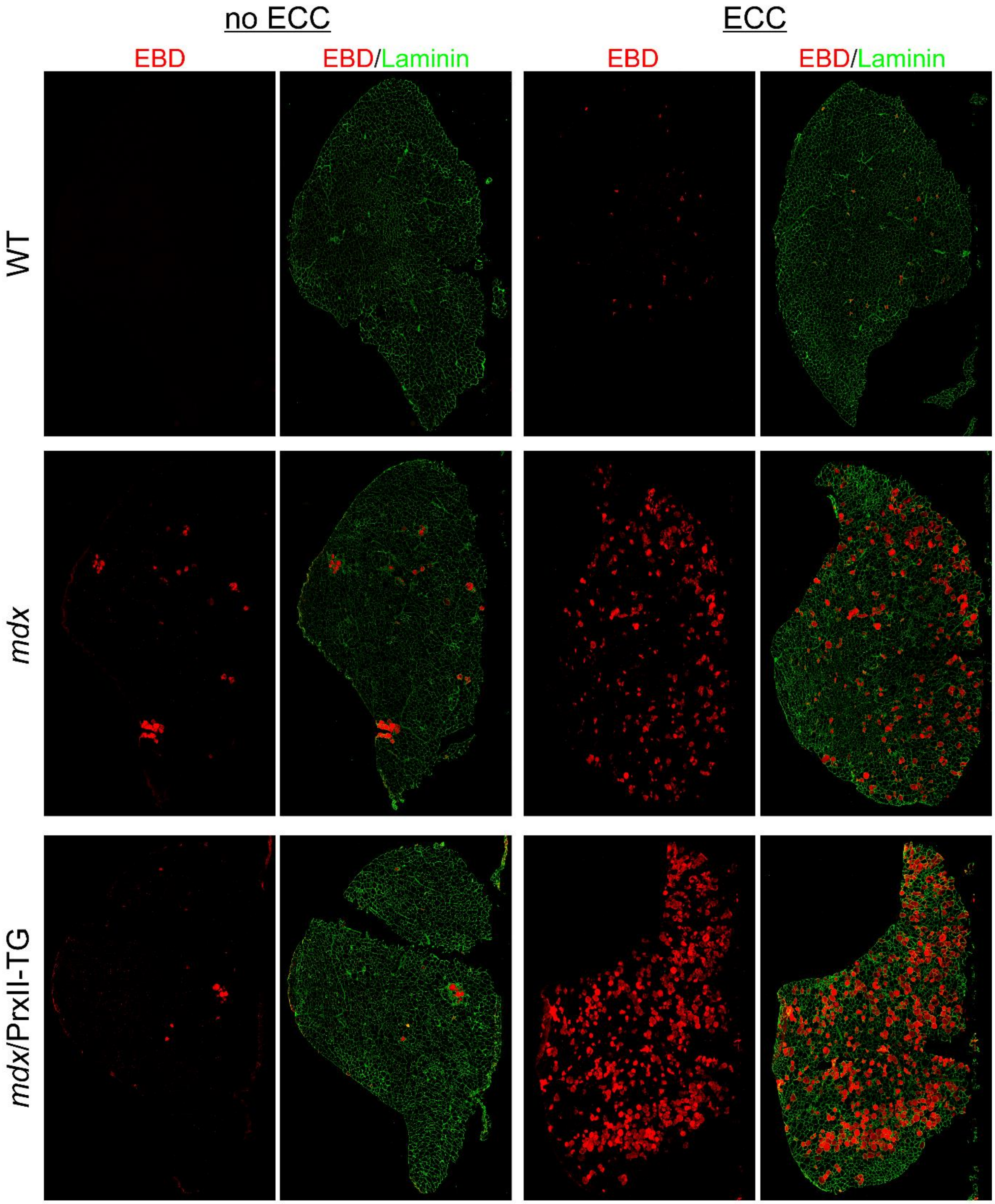

Supplementary Figure 7 Muscle-specific peroxiredoxin-2 overexpression leads to increased sarcolemmal damage following long-term injurious eccentric contractions in *mdx* mice. Fluorescent microscopy of WT, *mdx*, and *mdx*/PrxII-TG (58X) for Evans blue dye (EBD; red) and laminin (green). 10X images were stitched to allow visualization of the entire TA muscle in each genotype. no ECC = contralateral TA not subjected to eccentric contractions. ECC = TA subjected to 70 eccentric contractions performed *in vivo*.

Fig. 2a

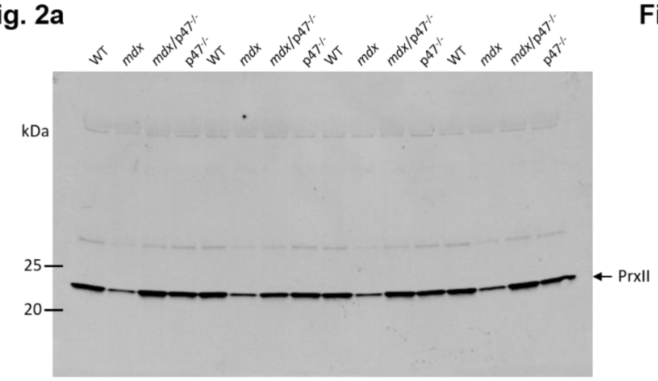

Fig. 2d

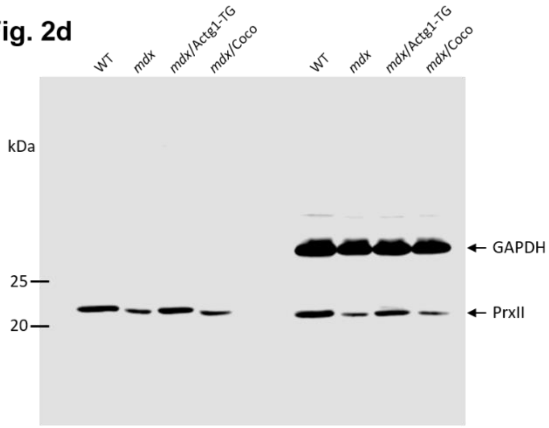

Fig. 2e

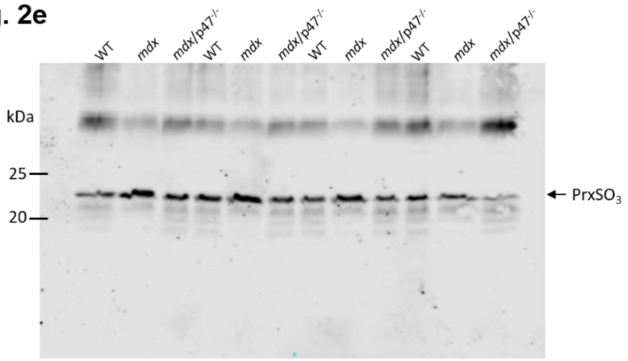

Supplementary Figure 8. Uncropped western blots of PrxII and $PrxSO_3$ from Figure 2.

Supplementary Table 1 Physiological parameters of isolated EDL muscles used in *ex vivo* force measurements.

Parameter	WT	*mdx*	*mdx*/ Actg1-TG	*mdx*/ Coco	*mdx*/ C272A-TG	*mdx*/ Actb-TG	*mdx*/$p47^{-/-}$	*mdx*/$mb^{-/-}$	*mdx*/$PrxII^{-/-}$	*mdx*/ PrxII-TG[@]	*P* value[$]
N	6	6	5	5	6	5	7	8	6	5	---
EDL mass (g)	13.0 ± 0.4	19.9 ± 0.8[*]	13.4 ± 1.1[#]	18.0 ± 0.9[*]	17.6 ± 0.5[*]	14.9 ± 0.5[#]	15.9 ± 0.2[#]	21.1 ± 1.2[*]	19.8 ± 0.7[*]	15.6 ± 0.7[#]	< 0.001
L_o (mm)	12.6 ± 0.1	13.4 ± 0.2	11.9 ± 0.3[#]	12.8 ± 0.4	13.9 ± 0.1[*]	12.3 ± 0.1[#]	12.5 ± 0.2	12.5 ± 0.1	14.1 ± 0.3[*]	12.7 ± 0.1	< 0.001
CSA (μm^2)	2.2 ± 0.1	3.1 ± 0.2[*]	2.4 ± 0.2	3.0 ± 0.2	2.7 ± 0.1	2.6 ± 0.1	2.7 ± 0.1	3.6 ± 0.2[*]	3.0 ± 0.1[*]	2.6 ± 0.1	< 0.001
Passive stiffness (N/m)	11.9 ± 0.4	17.9 ± 0.7[*]	14.4 ± 0.4	15.0 ± 0.4	14.7 ± 0.5	15.2 ± 0.7	14.7 ± 0.8	15.4 ± 0.9	16.7 ± 0.7[*]	16.7 ± 1.5[*]	< 0.001
Peak twitch (mN)	165 ± 15.9	101.1 ± 5.2[*]	116.3 ± 8.5[*]	109.8 ± 8.6[*]	108.5 ± 6.4[*]	118.5 ± 5.1	118.2 ± 6.3[*]	117.9 ± 13.1[*]	111.7 ± 7.3[*]	105.9 ± 3.3[*]	< 0.01
P_o (mN)	396.8 ± 7.2	383.4 ± 16.7	345.5 ± 16.6	393.4 ± 28.6	372.4 ± 12.9	386.7 ± 13.5	313.6 ± 16.6	422.4 ± 33.5	391.6 ± 12.9	342.7 ± 14.7	< 0.05
Specific P_o (N/cm^2)	17.9 ± 0.2	11.0 ± 0.3[*]	11.5 ± 0.5[*]	13.2 ± 1.1[*]	13.7 ± 0.4[*]	14.9 ± 0.3[#]	11.5 ± 0.7[*]	11.6 ± 0.5[*]	13.2 ± 1.0[*]	14.7 ± 0.5[#]	< 0.001
ΔP_o (%)	7.0 ± 1.8	87.4 ± 1.5[*]	77.0 ± 2.1[*]	81.1 ± 2.9[*]	88.5 ± 1.3[*]	82.3 ± 2.5[*]	81.2 ± 2.8[*]	74.3 ± 2.4[*#]	89.3 ± 0.5[*]	60.5 ± 4.8[*#]	< 0.001
Eccentric force loss (%)	2.0 ± 1.5[*]	83.3 ± 1.4[*]	69.0 ± 6.9[*]	80.6 ± 4.0[*]	83.4 ± 0.6[*]	68.0 ± 5.4[*#]	77.8 ± 2.2[*]	74.2 ± 1.5[*]	85.2 ± 1.4[*]	54.6 ± 5.1[*#]	< 0.001

[*]Significantly different from WT (Tukey post-hoc test).
[#]Significantly different from *mdx* (Tukey post-hoc test).
[$]One-Way ANOVA *P* value.
[@]58X PrxII-TG line.

Supplementary Table 2 Primers used in this study.

Primers	Sequence
PrxII Forward (qPCR)	5’-GGTTTGGGCCACGCATAAAA-3’
PrxII Reverse (qPCR)	5’-GCCATGACTGCGTGAGCAAG-3’
HPRT Forward (qPCR)	5’-CCCTGGTTAAGCAGTACAGCCCC-3’
HPRT Reverse (qPCR)	5’-GGCCTGTATCCAACACTTCGAGAGG-3’
HSA Forward (genotyping)	5’-GTCAGGAGGGGCAAACCCGC-3’
HSA Reverse (genotyping)	5’-GTCGCTGCCCTTCTCGAGCC-3’
