# Supplementary material for: Loss of peroxiredoxin-2 exacerbates eccentric contraction-induced force loss in dystrophin-deficient muscle
Source: Nat Commun. 2018 Nov 30;9:5104. doi: 10.1038/s41467-018-07639-3 (PMC6269445; doi:10.1038/s41467-018-07639-3)
Supplement: Supplementary file 4 — Description of Additional Supplementary Files [file 41467_2018_7639_MOESM4_ESM.docx]

**Title:** Supplementary Data 1

**Description:** Initial identification of proteins found in iTRAQ proteomic screen. Each mdx and mdx/Actg1-TG tibialis anterior sample was labeled with a unique iTRAQ tag shown above the protein list. Protein names and Accession Numbers are listed, with each iTRAQ-labeled sample taken as a ratio of tag 113 (mdx).

**Title:** Supplementary Data 2

**Description:** Differential protein list derived from iTRAQ proteomic screen with 1% local FDR analysis. All mdx and mdx/Actg1-TG iTRAQ-labeled samples were taken as a ratio of tag 113 (mdx). Protein names and corresponding Accession Numbers are listed, with the differential log expression and log P-value compared to sample 113 shown on the right.
